# Canopy-Forming Seaweeds in Urchin-Dominated Systems in Eastern Canada: Structuring Forces or Simple Prey for Keystone Grazers?

**DOI:** 10.1371/journal.pone.0098204

**Published:** 2014-05-23

**Authors:** Caitlin Blain, Patrick Gagnon

**Affiliations:** Department of Ocean Sciences, Ocean Sciences Centre, Memorial University of Newfoundland, St. John's, Newfoundland and Labrador, Canada; McGill University, Canada

## Abstract

Models of benthic community dynamics for the extensively studied, shallow rocky ecosystems in eastern Canada emphasize kelp-urchin interactions. These models may bias the perception of factors and processes that structure communities, for they largely overlook the possible contribution of other seaweeds to ecosystem resilience. We examined the persistence of the annual, acidic (H_2_SO_4_), brown seaweed *Desmarestia viridis* in urchin barrens at two sites in Newfoundland (Canada) throughout an entire growth season (February to October). We also compared changes in epifaunal assemblages in *D. viridis* and other conspicuous canopy-forming seaweeds, the non-acidic conspecific *Desmarestia aculeata* and kelp *Agarum clathratum*. We show that *D*. *viridis* can form large canopies within the 2-to-8 m depth range that represent a transient community state termed “*Desmarestia* bed”. The annual resurgence of *Desmarestia* beds and continuous occurrence of *D. aculeata* and *A. clathratum*, create biological structure for major recruitment pulses in invertebrate and fish assemblages (e.g. from quasi-absent gastropods to >150 000 recruits kg^−1^ *D. viridis*). Many of these pulses phase with temperature-driven mass release of acid to the environment and die-off in *D. viridis*. We demonstrate experimentally that the chemical makeup of *D. viridis* and *A. clathratum* helps retard urchin grazing compared to *D. aculeata* and the highly consumed kelp *Alaria esculenta*. In light of our findings and related studies, we propose fundamental changes to the study of community shifts in shallow, rocky ecosystems in eastern Canada. In particular, we advocate the need to regard certain canopy-forming seaweeds as structuring forces interfering with top-down processes, rather than simple prey for keystone grazers. We also propose a novel, empirical model of ecological interactions for *D. viridis*. Overall, our study underscores the importance of studying organisms together with cross-scale environmental variability to better understand the factors and processes that shape marine communities.

## Introduction

Erect fleshy seaweeds are a dominant component of shallow, rocky benthic communities in polar, subpolar, and cold-temperate seas [1]–[3]. By creating vertical structure that modifies the light [4], [5] and hydrodynamic environments [6], [7], seaweeds provide substrate and food for benthic and pelagic organisms [7]–[9], ultimately modulating predator-prey interactions [10]–[12]. Because seaweeds generally contribute to increasing marine biodiversity [13]–[16], any factor that alters their abundance is likely to trigger bottom-up cascades [3], [17], [18].

A prime example of cold, marine benthic communities organized around the high productivity of erect, fleshy seaweeds is that of the western Antarctic Peninsula. There, three perennial species with contrasting morphologies in the order Desmarestiales, *Desmarestia anceps*, *Desmarestia menziesii*, and *Himantothallus grandifolius*, form thick canopies that cover up to 80% of the seabed [2], [3]. These foundation species [18], which, presumably, chemically deter dominant grazers [19]–[21], provide continuous access to vertical structure for the recruitment and growth of highly diverse assemblages of invertebrates [22]–[24]. This condition contrasts with shallow marine ecosystems at lower latitudes in both hemispheres, where macroherbivores largely control the structure and dynamics of benthic communities [25]–[27].

Empirical and analytical models of benthic community dynamics for the extensively studied, shallow rocky ecosystems in eastern Canada (Nova Scotia and northwards) emphasize kelp-urchin interactions. These models often include shifts in the distribution and abundance of kelp (mainly *Saccharina longicruris* and *Alaria esculenta*) that can be predicted reasonably well from population shifts in their main predator, the omnivorous green sea urchin, *Strongylocentrotus droebachiensis* [28]–[30]. The traditional view that these ecosystems exhibit alternations between two community states, kelp bed and urchin barrens, was recently broadened. It includes, for the Nova Scotia region, a likely transient (multiyear) state dominated by the introduced green seaweed *Codium fragile* ssp. *fragile* [31], [32]. The latter state, together with improving knowledge about the stability and functional importance of less studied, canopy-forming seaweeds in urchin barrens [11], [28], [30], [33], [34], call for a critical reassessment of the generality of phase-shifts and their mechanisms.

Recent studies of acid (H_2_SO_4_) production and mortality in the annual Desmarestiales *Desmarestia viridis* in urchin barrens in Newfoundland show that the acid continuously and irreversibly accumulates within vacuoles as sporophytes grow from recruit to adult (March-June). This build-up lowers the intracellular pH to 0.53 and inevitably culminates into mass releases of acid (July-August) and die-offs (September-October), when mean sea temperature rises above 12°C [35], [36]. These and other studies in the northern Gulf of St. Lawrence [11], [28] and Nova Scotia ([30], [37], P. Gagnon unpublished data) suggest that *D. viridis* may limit urchin movement and facilitate recruitment in invertebrates and other seaweeds, including kelp. The notion that *D. viridis* contributes to the development of an ephemeral community state has yet to be scrutinized with integrated studies of the persistence, functional importance, and mechanisms that promote the survival of sporophytes in urchin barrens.

In the present study, we test the overall hypothesis that *D. viridis* functions as an ephemeral foundation species facilitating recruitment in, and supporting distinct assemblages of, invertebrates in urchin barrens at two subtidal sites in Newfoundland. Specifically, we 1) characterize and relate temporal variability in the abundance of *D. viridis* and green sea urchins at multiple depths, as well as 2) compare changes in epifaunal assemblages of *D. viridis* and two other conspicuous, canopy-forming seaweeds in urchin barrens, the non-acidic Desmarestiales *Desmarestia aculeata* and the grazing-resistant kelp *Agarum clathratum* throughout an entire growth season (February to October, 2011) in *D. viridis*. We also 3) conduct two complementary laboratory experiments to assess the vulnerability of *D. viridis*, *D. aculeata*, and *A. clathratum* to grazing by the green sea urchin and how it relates to the seaweed chemical makeup. In light of our findings and related studies, we propose fundamental changes to the study of community shifts in shallow rocky ecosystems and a novel empirical model of ecological interactions for *D. viridis* in eastern Canada.

## Materials and Methods

### Study sites and characteristics of seaweeds studied

**Figure 1:**
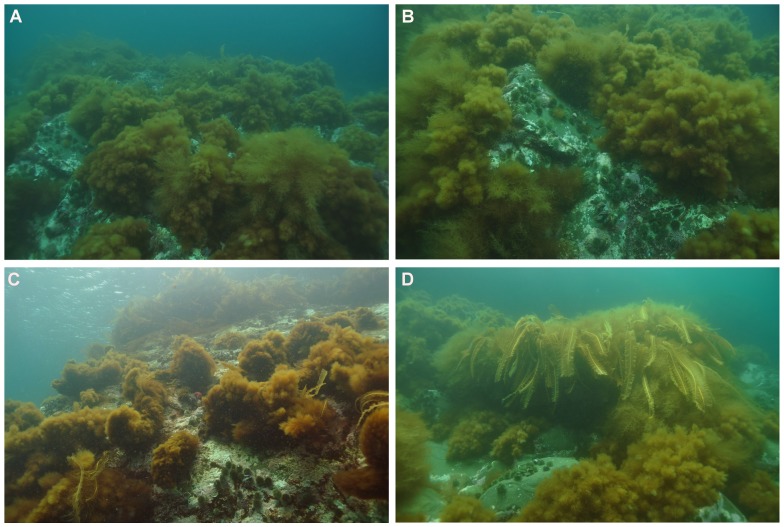
A thick canopy (∼70–90% cover) of sweeping *Desmarestia viridis* sporophytes at depths of 5 to 8 m on urchin (*Strongylocentrotus droebachiensis*) barrens in Bay Bulls (Newfoundland, eastern Canada) in June 2012 (A) and July 2013 (B). Maturing sporophytes of *Desmarestia aculeata* (foreground) and the kelp *Alaria esculenta* (background) intersperse with the *D. viridis* canopy. A sparse canopy (∼10–15% cover) of *D. viridis* below the lower edge of a shallow (0–2 m deep) *A. esculenta* bed in June 2011 (C). A cluster of *A. esculenta* amidst mixed canopy of *D. viridis* and *D. aculeata* at a depth of ∼3 m in July 2011 (D). Note that urchins are largely restricted to non-swept rocky surfaces (A, B, C, and D). (Photos: Patrick Gagnon)

This study was conducted with *Desmarestia viridis*, *Desmarestia aculeata*, *Agarum clathratum*, *Alaria esculenta*, and green sea urchin at, or collected from, two gently sloping, rocky subtidal sites located ∼1.4 km apart in Bay Bulls, on the southeastern tip of Newfoundland: Bread and Cheese Cove (BCC, 47°18’35’’ N, 52°47’30’’ W) and Keys Point (KP, 47°18’15’’ N, 52°48’24’’ W). All necessary permits for sampling and collecting seaweeds and urchins were obtained prior to sampling in accordance with the Canadian Council of Animal Care guidelines. No specific locational permits were required for seaweed and urchin sampling and collection in Bay Bulls, and no threatened or endangered species were at risk of incidental capture. Seaweed assemblages at both sites are dominated by the kelps *Alaria esculenta* and *Laminaria digitata* to a depth of ∼2 m, followed by extensive pavements of red coralline seaweeds, mainly *Lithothamnion glaciale*, to a depth of ∼15 m. These pavements, hereafter termed “barren zone” or “barrens” to follow the convention, are colonized year round by the green sea urchin, *Strongylocentrotus droebachiensis*, as well as *D. aculeata*. The latter forms small (a few m^2^), scattered patches on boulder tops and ridges at depths between 2 and 10 m. *Desmarestia viridis* sporophytes establish annually in both barrens from March to October [35] (Fig. 1). The grazing-resistant kelp *A. clathratum* [34] forms small, scattered patches throughout the barrens and large (up to several tens of m^2^) stands at depths >15 m.

Of the three seaweeds in urchin barrens that we investigated for epifaunal assemblages (see below), only one, *D. viridis*, has an annual life cycle. Sporophytes exhibit three phases of change in length at BCC and KP: (1) increase [March to late June], (2) no change [July to mid-August], and (3) decrease [mid-August to late October] [35]. The two other species, *D. aculeata* and *A. clathratum*, are perennials with sporophytes that can live up to at least a few years [38], [39] and, in the case of *A. clathratum*, form highly stable patches in urchin barrens [34]. Sporophytes in both *D. viridis* and *D. aculeata* have a highly flexible stipe and a profusely branched frond, which sweeps back and forth over the bottom with wave action [11], [40]. This effect is less pronounced in *D. aculeata* because of cortication, during summer, of new tissues added annually [38], [41]. Sporophytes of *A. clathratum* have a semi-rigid stipe and a large crinkled frond, which becomes thicker and tougher with age [39], thereby limiting frond movement compared to *D viridis* and *D. aculeata*. The relatively low palatability of *D. viridis* and *A. clathratum* to urchins is, presumably, due to chemical deterrents in frond tissues, sulfuric acid and phenolics, respectively [42]–[45]. There is no known anti-grazing substance in *D. aculeata* [44], [46], [47].

### Distribution and abundance of *D. viridis* and urchins

To evaluate the variability in the distribution and abundance of *Desmarestia viridis* and green sea urchin, we monitored changes in the cover (*D. viridis*) and density (urchin) in the barrens at BCC and KP during the entire 2011 sporophyte growth season in *D. viridis*. At each site, we permanently marked, in January 2011, both ends of one 20- to 25-m benchmark line running parallel to the shoreline at 2, 3, 4, and 8 m depths with bolts set into the bedrock. These depths covered the vertical range of *D. viridis* at both sites based on surveys in previous years. We sampled the 2-to-4 m range more intensively because this is where *D. viridis* and urchin abundances were more likely to vary in response to generally more variable temperature and wave conditions than in deeper (8 m) water [35], [36]. On 8 March, 2011, a 1-m swath of seabed was filmed on each side of each benchmark line with a submersible, digital video camera (Sony HVR-V1 with an Amphibico Endeavor housing) propelled by a diver at a speed of ∼0.1 m sec^−1^ at a distance of 1.5 m above the bottom. This procedure, which yielded two video transects per depth, was repeated biweekly until 13 October, 2011 after which all *D. viridis* sporophytes had deteriorated to a point where they were too small (∼1 cm) to be detected on the imagery. All sporophytes had disappeared by 20 October [35].

Each video transect was then converted into one image strip with PanoraGen.DV V1.0. Depending on clarity, each image was segmented into 12 to 25 frames of 0.8 m^2^ with PhotoImpact V6.0. The percentage cover of *D. viridis* was estimated within each of five randomly selected frames using a digital grid with 100 point intersects, for a total of 10 cover estimates per depth. Urchin density was obtained by dividing the number of urchins >1 cm in test diameter (the smallest detectable size on the imagery) in each of five haphazardly selected frames without fleshy seaweeds, by the surface area of the frame, also yielding 10 density estimates per depth. For urchin density, we worked only with those frames without seaweeds because it is the urchins in areas of the barrens devoid of erect fleshy seaweeds (open areas) that can affect the cover of *D. viridis*. Indeed, it is well established that urchins largely avoid venturing underneath sweeping *D. viridis* sporophytes (e.g. [11], [40]). Urchins concentrate in open areas until wave action is too low to induce sweeping in *D. viridis*. When wave action is sufficiently low, urchins move towards non-sweeping sporophytes and aggregate at their periphery. Urchins graze the apical parts of the fronds until wave action and sweeping resume, which forces urchins to return to open areas. The few urchins underneath *D. viridis* are basically clinging to the substratum.

### Epifaunal assemblages

To assess the functional importance of *Desmarestia viridis*, *Desmarestia aculeata*, and *Agarum clathratum*, we tracked changes in epifaunal assemblages of each species at KP during the entire 2011 growth season in *D. viridis*. Given 1) interspecific differences in ontogeny, morphology, and chemistry of sporophytes outlined above, and 2) marked changes in length, bushiness, and intracellular acidity of *D. viridis* sporophytes within only a few months [35], [36], we predicted that epifaunal assemblages in *D. viridis* would be more variable and less diverse than those in the longer-lived, morphologically comparable, non-acidic *D. aculeata*. We also predicted that epifaunal assemblages would be more variable and diverse in *D. viridis* than in the perennial and morphologically less complex *A. clathratum*.

On 18 February, 2011 and every 26 to 35 days until 9 October, 2011 we hand collected (via SCUBA diving) frond tissues from 7 to 10 sporophytes in each seaweed at depths between 6 and 10 m (*D. aculeata*) and 8 and 12 m (*D. viridis* and *A. clathratum*). It was not possible to collect the three seaweeds within a common depth range. Yet, less than 20% of the sporophytes were located in slightly shallower (2 m) water than the rest of the sporophytes. We did not perceive any marked changes in environmental conditions across the 6-to-12 m range. Therefore, we assumed that the effect of depth on epifaunal assemblages, if any, was unlikely to overshadow that of seaweed identity. Tissues from only those sporophytes that showed no, or the least pronounced, external signs of deterioration were collected (frond discoloration and sloughing in *D. viridis* began at the shallower depths in late July). Approximately 10 g of tissues (representing ∼5% of the wet weight of the largest sporophytes in July) were cut with scissors from the distal end of each sporophyte. Tissues were placed immediately in rigid 4-L plastic containers (one piece per container) sealed under water to prevent the loss of epifauna. Containers were transported to the Ocean Sciences Centre (OSC) of Memorial University of Newfoundland where the content of each container was sieved. Each piece of seaweed was gently groomed to ensure all epifauna ≥250 µm were collected. Encrusting or gelatinous invertebrates such as bryozoans, cnidarians, and egg masses were identified and counted (individuals or colonies) within 24 hours from arrival at the OSC. All other epifauna were immersed in a 5% formalin-seawater solution for 24 hours and transferred into glass vials with a 70% ethanol-freshwater solution for preservation and later identification and counting. Organisms were identified to species where practical, and to genus (e.g. *Mytilus* sp.) or family (e.g. Halacaridae) otherwise. Seaweed tissue wet weight was determined with a balance (±0.01 g, model PB-3002-S/FACT; Mettler Toledo).

### Vulnerability to grazing

To assess the vulnerability of *Desmarestia viridis*, *Desmarestia aculeata*, and *Agarum clathratum* to grazing and how it relates to the seaweed chemical makeup, we conducted two complementary experiments with intact sporophyte tissues (Experiment 1) and gelatinized extracts of grinded sporophyte tissues (Experiment 2). We used the green sea urchin as the grazer since it is the dominant consumer of the three seaweeds in eastern Canada [11], [34], [48], including at our study sites. Experiment 1 tested the hypothesis that in the absence of waves and currents, vulnerability (tissue loss) to grazing of *A. clathratum* (phenolics) is lower than that of *D. viridis* (sulfuric acid), which in turn is lower than that of *D. aculeata* (no known chemical deterrent). We used the kelp *Alaria esculenta*, which is one of the most consumed seaweeds by *S. droebachiensis* in the northwestern Atlantic (NWA) [42], [48], to test the validity of the results, with the expectation that vulnerability is highest in *A. esculenta*. We ran the experiment from 15 to 19 July, 2011 with tissues from five sporophytes in each species collected (via SCUBA diving) on 14 July at depths between 2 and 15 m at KP. One piece of ∼30 g of tissues was cut with scissors from the apical parts (*D. viridis* and *D. aculeata*) or edges of the frond (*A. clathratum* and *A. esculenta*) of each sporophyte because in natural habitats urchins typically contact and consume these portions first. Pieces were placed in rigid, 4-L plastic containers (one piece per container) sealed under water to prevent contact of tissues with air at the surface. Tissues from only those sporophytes that showed no, or the least pronounced, external signs of deterioration were collected. Containers were transported to the OSC where their content was transferred to large holding tanks supplied with ambient (7.8±0.5°C), flow-through seawater pumped in from the adjacent embayment, Logy Bay.

On 15 July (within less than 24 hours of seaweed collection), we cut two pieces of ∼10 g (wet weight) from each original sample of ∼30 g. Tissue weight was determined with precision with a balance (same model as above) in less than 20 s following emersion. Such inevitable exposure of *D. viridis* tissues to air had no effect on acidity [35]. One piece of each pair was transferred to either of 20, 75-L glass tanks supplied with flow-through sea water (1 L min^−1^), and secured to the bottom with 12-g weights attached to the stipe or frond with a plastic cable tie. Ten urchins (3 to 6 cm in test diameter) picked from a pool of individuals collected on 7 July, 2011 at KP and starved for one week to standardize hunger levels, were introduced to each tank and allowed to graze seaweeds for 48 hours. Urchin density in each tank was generally equivalent to that in the barrens at our two study sites. Each piece of seaweed was reweighed at the end. Tissue loss to grazing was corrected for autogenic loss or gain, as determined by applying the procedures above to the second set of 10-g pieces of sporophytes over the following 48 hours, except no urchins were introduced to the tanks. We used the following equation to obtain the corrected tissue loss in each tank [49]:
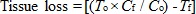
where *T*_o_ and *T*_f_ are the initial and final weights of seaweed tissues exposed to urchins, respectively, and *C*_o_ and *C*_f_ are the mean initial and final weights of the corresponding autogenic control, respectively.

The 20 tanks were grouped in five blocks of four tanks. Each tank in each block was randomly assigned one of the four seaweeds, for a total of five replicates per treatment. Each tank was surrounded by opaque canvas to control light conditions. Standardized light intensities were created with an incandescent, 100-watt light bulb (Soft White, General Electric) located at 45 cm above the water surface and controlled with dimmers on a 12-hour light/dark cycle. To increase the sample size, we reran the experiment, including the autogenic controls, twice from 19 to 23 and 23 to 27 July, 2011. We used tissues from two groups of 20 sporophytes (five in each of the four species in each group) collected on 18 and 22 July, and urchins collected on 12 and 14 July starved for one week. As in the first run, treatments in each of the two additional runs were reassigned randomly to tanks in each block to eliminate confounding effects of treatment and block, as well as tank and treatment. Therefore, each treatment was replicated 15 times in total. Temperature in one randomly chosen tank of each treatment was monitored with a temperature logger (±0.5°C, HOBO Pendant; Onset Computer Corporation) throughout the experiment.

Experiment 2 examined the unique contribution of seaweed chemical makeup to grazer deterrence. It also provided an indirect test for urchin-perceived differences in non-chemical traits among seaweeds in Experiment 1, including tissue toughness, which cannot be separated from the chemical makeup without altering the structural integrity of tissues. Experiment 2, therefore, tested the hypothesis that vulnerability to grazing depends primarily on the chemical makeup, with the same expectation in species ranking than in Experiment 1. As in Experiment 1, we used *A. esculenta* as a reference with the expectation that it is the most vulnerable of the four species. We ran the experiment from 27 to 31 July, 2011 with tissues from four sporophytes in each species collected (via SCUBA diving) on 25 July at depths between 2 and 15 m at KP. Procedures for collection, transportation, and maintenance of sporophytes in the laboratory were similar to those in Experiment 1. Water temperature during acclimation in the holding tanks was 8.8±0.5°C.

On 27 July (within less than 48 hours of seaweed collection), we cut one piece of ∼10 g (wet weight, measured with the same balance as above) from each original sample of ∼30 g. Each piece weighed from 9.8 to 10.2 g and was crushed for 60 s in 100 mL of distilled water with a high-speed blender (model Magic Bullet; Homeland Housewares). The blend was suctioned through a 25-µm filter paper (model 1004-070; Whatman) to remove particulates. Eighty (80) mL of the filtrate (extract) were agitated and warmed on a heating plate (model VMS-C7 S1; VWR International) after adding 2 g of granulated agar (product BP1423-500; Fisher Scientific) to it. The resulting extract-agar solution was poured into two circular, 50-mL Petri dishes and allowed sufficient time to cool. Each solidified disk was removed from its dish and weighed with a balance (same model as above). We prepared eight additional disks, each made up of 40 mL of distilled water and 1 g of agar, to verify that urchins consumed, and hence were not repelled by, the raw agar medium without seaweed extracts (procedural control). One disk of each pair with seaweed extracts, and four disks with no extracts, were each transferred to one of 20, 75-L glass tanks supplied with flow-through sea water (1 L min^−1^) and secured to the bottom with 5-g weights. Ten urchins (3 to 6 cm in test diameter) picked from a pool of individuals collected on 18 July, 2011 at KP and starved for one week, were introduced to each tank and allowed to graze disks for 48 hours. Each disk was reweighed at the end. Disk loss to grazing was corrected for artificial loss or gain as determined by applying the procedures above to the second set of disks over the following 48 hours in the absence of urchins. We used the same equation as in Experiment 1 to obtain the corrected disk loss in each tank.

The 20 tanks were grouped in four blocks of five tanks. Each tank in each block was randomly assigned one of the five treatments, for a total of four replicates per treatment. Light conditions in each tank were similar to Experiment 1. To increase the sample size, we reran the experiment, including the procedural and autogenic controls, four times between 31 July and 20 August, 2011. We used tissues from four groups of 16 sporophytes (four in each of the four species) and urchins collected <72 hours and <10 days prior to each run, respectively. As in the first run, treatments in each of the four additional runs were reassigned randomly to tanks in each block to eliminate confounding effects of treatment and block, as well as tank and treatment. Therefore, each treatment was replicated 20 times in total. Temperature in one randomly chosen tank of each treatment was also monitored with a temperature logger (same model as above) throughout the experiment. Mean water temperature during trials was comparable between Experiment 1 (6.1±0.2°C) and Experiment 2 (8.3±0.1°C).

### Statistical analysis

We used a three-way ANOVA with the factors Site (two study sites: BCC and KP), Depth (four sampling depths: 2, 3, 4, and 8 m), and sampling Date (12 sampling dates from 8 April to 23 September, 2011), to examine effects of site, depth, and time on the mean cover of *Desmarestia viridis*. Data acquired in March and October were excluded from the analysis (these data are nevertheless presented graphically) because mortality of *D. viridis* sporophytes in these months is highly variable and largely affected by factors other than urchin grazing [35]. Site was a fixed factor because the two sites were selected specifically for their locations (opposite sides of Bay Bulls), and bathymetry (gently sloping, rocky substrata). The analysis was applied to the raw data on individual frames taken from each pair of video transects at each depth (2 sites × 4 depths × 12 dates × 2 transects × 5 frames; n = 960). F-ratios were formed according to expected mean squares, as in Quinn and Keough [50]. We partitioned the error term into among and within transect variation and formed the F-ratios of the main effects and their interactions over the MS error among transect to avoid confounding effect of transects (Table 1) [50]. We then used simple linear regression analysis [51] to relate *D. viridis* cover to urchin density for each depth at each site. Each regression model (eight in total) was based on 12 data points. Each point was the mean *D. viridis* cover and corresponding mean urchin density calculated from the 10 frames of each pair of transects for a given site, depth, and date. All regressions were applied to the raw data.

**Table 1:** Summary of three-way ANOVA (applied to raw data) examining the effect of Site (BCC and KP), Depth (2, 3, 4, and 8 m), and sampling Date (12 dates) on the cover of *Desmarestia viridis* on the seabed from 8 April to 23 September, 2011 (see “Materials and methods” for the details of the two error terms).

Source of variation	*df*	SS	MS	*F*-value	*P*
Site	1	2760.48	2760.48	130.43	<0.0001
Depth	3	6385.47	2128.49	100.57	<0.0001
Date	11	6162.65	560.24	26.47	<0.0001
Site × Depth	3	2121.58	707.19	33.42	<0.0001
Site × Date	11	1998.81	181.71	8.59	<0.0001
Depth × Date	33	6847.43	207.50	9.80	<0.0001
Site × Depth × Date	33	3607.22	109.31	5.16	<0.0001
Error (among transect)	96	2031.71	21.16	0.19	
Error (within transect)	768	84192.02	109.63		
Corrected total	959				

Values for density of epifaunal taxa on *D. viridis*, *Desmarestia aculeata*, and *Agarum clathratum* were 4^th^-root transformed prior to multivariate analysis because of differences of up to three orders of magnitude within and among the months sampled (February to October). We used non-metric multidimensional scaling (nMDS) based on Bray-Curtis similarity matrices calculated from these transformed data to visualize distances among epifaunal assemblages [52]. Inspection of the nMDS plot (see Results) from all monthly averages of samples in the three seaweeds [n = 7 to 10, except 3 for *A. clathratum* in February, for a total n = 221] directed the following approach to data analysis. We used a one-way analysis of similarity (ANOSIM) with the factor Seaweed (*D. viridis*, *D. aculeata*, and *A. clathratum*) to test for significant differences in assemblages among the three seaweeds (data pooled across all months, separated by seaweed) [52]. We also used a one-way ANOSIM with the factor Group (1 [data pooled across seaweeds for February to August] and 2 [data pooled across seaweeds for September and October]) to test for significant differences in assemblages between the first seven and last two months. The latter two ANOSIMs yielded significant differences, which we further investigated by analyzing each seaweed separately. Inspection of the nMDS plot of each seaweed from monthly samples (n =  80 [*D. viridis*], 77 [*D. aculeata*], and 64 [*A. clathratum*]) also directed the use of a one-way ANOSIM to test for significant differences in assemblages between Group 1 and Group 2 (same grouping as above). This was followed by a one-way analysis of similarity percentage (SIMPER) in each of Group 1 and Group 2 to assess the degree of similarity of assemblages among sampling events. Lastly, we used two one-way SIMPER analyses with the factor Seaweed (*D. viridis*, *D. aculeata*, and *A. clathratum*) to identify taxa important in distinguishing the assemblages associated with each seaweed, one for February to October (entire duration of the study) and one for September and October (when epifaunal abundance and diversity changed markedly, see Results).

We used two-way ANOVAs (one for each group of organisms) with the factors Seaweed (three seaweeds: *D*. *viridis*, *D*. *aculeata*, and *A*. *clathratum*) and Month (nine levels: February to October) to examine temporal changes within and between seaweeds in the density of individuals in the six numerically dominant (i.e. with a peak density ≥900 individuals kg^−1^ seaweed in any month) invertebrate taxa (Bivalvia, Gastropoda, Copepoda, Amphipoda, Polychaeta, and Isopoda), and fish and gastropod egg masses. Raw sample sizes for each seaweed in each month ranged from 7 to 10, except 3 for *A. clathratum* in February. No transformation corrected the heteroscedasticity of the residuals in the eight analyses on the raw data (n = 221). Therefore, the ANOVAs were also run with the rank-transformed data. Because analyses on both raw and ranked-transformed data gave similar conclusions about the significance of each factor, we presented the results from analyses of the raw data, as suggested by Conover [53]. Likewise, we used three two-way ANOVAs with the factors Seaweed (three seaweeds: *D*. *viridis*, *D*. *aculeata*, and *A*. *clathratum*) and Month (nine levels: February to October) to examine temporal changes in the Shannon diversity index (*H'*), Pielou's evenness index (*J'*), and species richness (S) of epifauna. The latter three analyses were applied to the raw data (n = 221).

We used a one-way ANOVA with the factor Seaweed (four seaweeds: *D. viridis*, *D. aculeata*, *A. clathratum*, and *Alaria esculenta*) to examine differences in the proportion of seaweed tissue weight loss (relative to initial weight) to urchin grazing (Experiment 1). Likewise, we used a one-way ANOVA with a similar structure and a fifth level (the procedural control) within the factor Seaweed, to examine differences in the proportion of agar-embedded seaweed extracts weight loss (relative to initial weight) to urchin grazing (Experiment 2). We treated both analyses as a particular case of the generalized linear models with a binomial distribution of the response variable (ratio of final to initial weigh) [54], [55]. No binomial variation was detected. Prior to running these one-way ANOVAs, we used two three-way ANOVAs with the additional factors Run (each of three or five runs of replicates in Experiment 1 and Experiment 2, respectively) and Block (each of five or four blocks of tanks in each run in Experiment 1 and Experiment 2, respectively), to determine whether results differed between runs and blocks. There was no significant interactions between the factors Run and Block in both analyses (Factor  =  Run x Block, χ^2^ = 2.12, p = 0.98 [Experiment 1]; χ^2^ = 0.78, p = 0.99 [Experiment 2]). Therefore, we applied the two one-way ANOVAs to the pooled data from all runs in each experiment.

In all ANOVAs and regression analyses, homogeneity of the variance was verified by examining the distribution of the residuals. Normality of the residuals was verified by examining the normal probability plot of the residuals [56]. To detect differences among levels within a factor, we used Tukey HSD multiple comparison tests (comparisons based on least-square means) [51]. A significance level of 0.05 was used. All analyses were conducted with JMP 7.0 and Minitab 16, except multivariate analyses, which were carried out with Plymouth Routines in Multivariate Ecological Research (PRIMER) v6.1.10.

## Results

### Distribution and abundance of *D*. *viridis* and urchins

**Figure 2:**
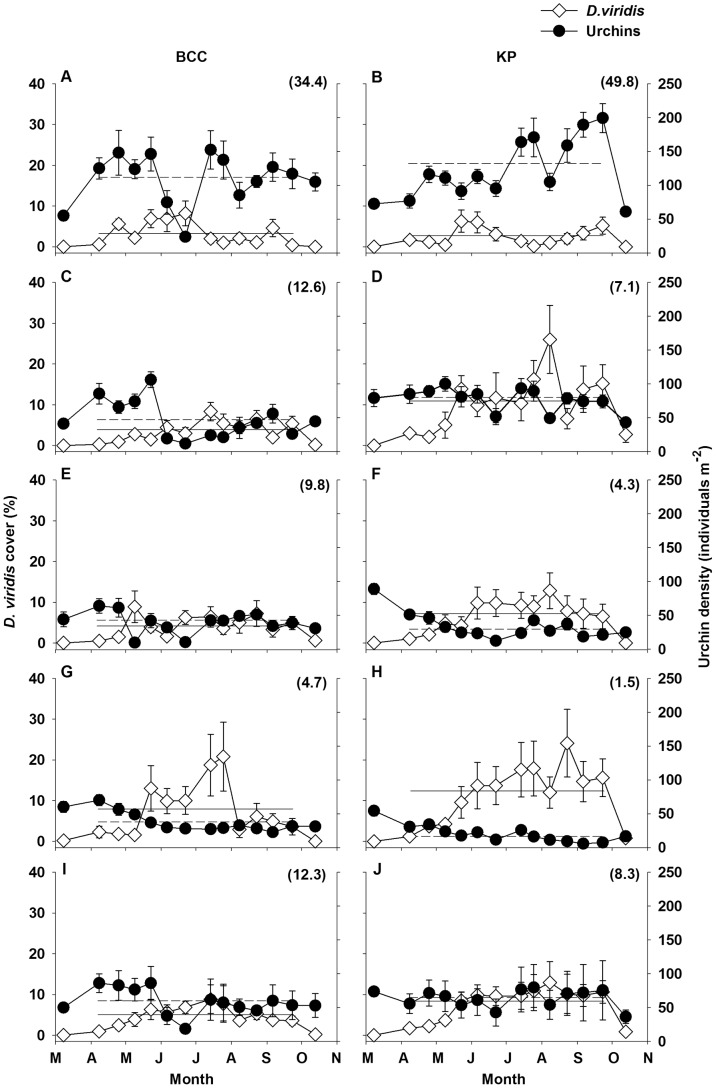
Mean (±SE) *Desmarestia viridis* cover and urchin (*Strongylocentrotus droebachiensis*) density at 2 (A, B), 3 (C, D), 4 (E, F), and 8 (G, H) m depths, and averaged across all depths (I and J) at Bread and Cheese Cove (BCC) and Keys Point (KP) from 8 March to 13 October, 2011. Each data point in panels A to H is the average cover of *D. viridis* or urchin density in 10 quadrats (0.8 m^2^ each) from two transects (20 to 25 m) at each depth. The seeming lack of standard error on some data points is due to low data variation. Solid and dashed horizontal lines are the average *D. viridis* cover and urchin density, respectively, from 8 April to 23 September (12 data points; see “Materials and methods” for the details of restriction of the statistical analyses to these points). The number in parentheses within each panel is the ratio of urchin density to *D. viridis* cover, also from 8 April to 23 September.

Patterns of *Desmarestia viridis* and urchin abundances at 2, 3, 4, and 8 m depths at BCC and KP from March to October, 2011 suggested a general increase in *D. viridis* cover and decrease in urchin density with increasing depth (Fig. 2). The highest mean cover of *D. viridis* ranged from 8% at 2 m to 21% at 8 m at BCC, and from 6% at 2 m to 25% at 8 m at KP. The highest mean urchin density ranged from 152 individuals m^−2^ at 2 m to 70 individuals m^−2^ at 8 m at BCC, and from 199 individuals m^−2^ at 2 m to 54 individuals m^−2^ at 8 m at KP (Fig. 2). In general, the overall cover (all depths pooled) of *D. viridis* at both sites: 1) increased steadily from March to mid-July; 2) decreased slowly to intermediate values from mid-July to late September; and 3) further decreased dramatically to extinction by mid-October (Table 1, Fig. 2). The latter decrease was when mean sea temperature peaked above 10°C [35] and urchins were below ∼75 individuals m^−2^ (Fig. 2). Yet, cover showed some differences between sites among depth, being generally higher at KP than BCC at all depths except at 2 m where it was similar between sites (Table 1, Fig. 2).

**Figure 3:**
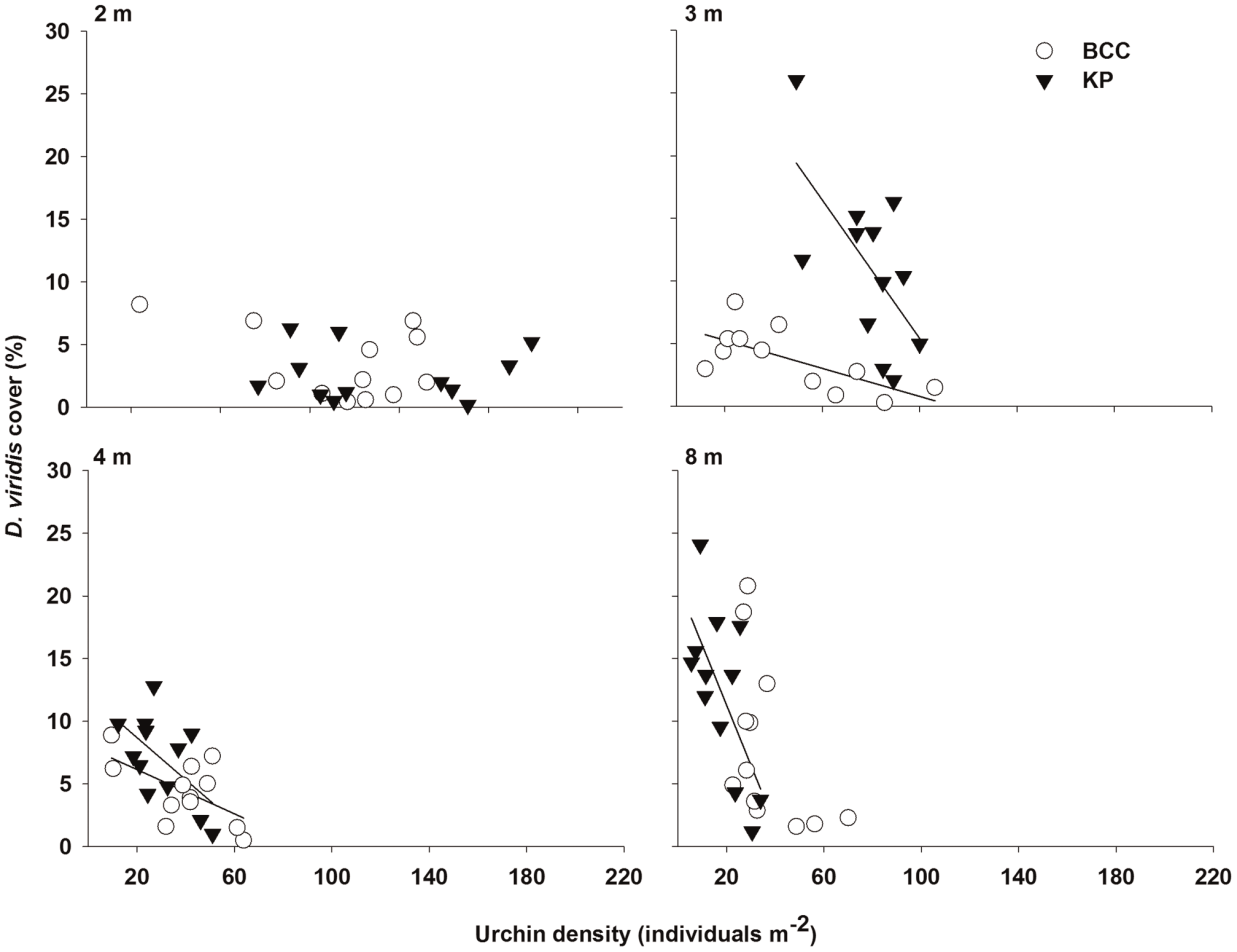
Relationships between *Desmarestia viridis* cover and urchin (*Strongylocentrotus droebachiensis*) density at 2, 3, 4, and 8 m depths at Bread and Cheese Cove (BCC) and Keys Point (KP) from 8 April to 23 September, 2011. Each data point is the mean cover and corresponding mean density calculated from the 10 frames of each pair of transects for a given site, depth, and date. Solid lines are the linear regression fits to data for each site (p<0.05; n = 12) (see Table 2 for the details of the regressions).

Regression analysis revealed significant, negative relationships explaining 33% to 48% of the variation between *D. viridis* cover and urchin density at 3 and 4 m at BCC, and at 3, 4, and 8 m at KP (no significant relationship for the other combinations of site and depth; Table 2, Fig. 3). The urchin density to *D. viridis* cover ratio is a proxy for *D. viridis* vulnerability to urchin grazing: the greater the ratio, the more urchins per unit of *D. viridis* cover, and hence higher the theoretical vulnerability [40]. This ratio was 1.4 times greater at KP (49.8) than BCC (34.4) at 2 m, yet between 1.8 and 3.1 times lower at KP than BCC at greater depths, i.e. a reverse difference between sites that increased from 3 to 8 m (Fig. 3). These results, together with the 1.5-fold greater overall ratio (all depths pooled) at BCC than KP (Fig. 3), suggest that the theoretical vulnerability of *D. viridis* to urchin grazing was generally higher and more uniform across depth at BCC. At both sites, urchin grazing on *D. viridis* from March to July was limited to only a few scattered days when wave action, and hence the wave-induced sweeping motion of sporophytes, was virtually null. However, we witnessed dramatic increases in urchin grazing on *D. viridis* in early August, which persisted, especially at greater depths, until complete disappearance of *D. viridis* in October.

**Table 2:** Results of simple linear regression analyses (applied to raw data) examining the relationship between *Desmarestia viridis* cover and urchin (*Strongylocentrotus droebachiensis*) density (x, urchins m^−2^) at 2, 3, 4, and 8 m depths at each of the two study sites, Bread and Cheese Cove (BCC) and Keys Point (KP) from 8 April to 23 September, 2011.

Site	Depth (m)	Equation for *D. viridis* cover (%)	*r* ^2^	*F*_(*df*)_	*p*
BCC	2	7.00–0.031 x	0.169	2.027_(1,10)_	0.19
	3	6.40–0.056 x	0.482	9.29_(1,10)_	0.012
	4	7.92–0.088 x	0.352	5.44_(1,10)_	0.042
	8	16.55–0.23 x	0.253	3.89_(1,10)_	0.096
KP	2	2.70–0.00028 x	<0.0001	<0.001_(1,10)_	0.99
	3	33.074–0.28 x	0.407	6.85_(1,10)_	0.026
	4	12.044–0.17 x	0.334	5.023_(1,10)_	0.049
	8	20.93–0.48 x	0.453	8.27_(1,10)_	0.017

Each regression is based on 12 data points. Each point is the mean *D. viridis* cover and corresponding mean urchin density calculated from the 10 frames of each pair of transects for a given site, depth, and date.

### Epifaunal assemblages

Epifaunal assemblages on *Desmarestia viridis*, *Desmarestia aculeata*, and *Agarum clathratum* from 18 February to 9 October, 2011 at KP consisted of 41 taxa; 38 invertebrates and three chordates (all juvenile fish), in eight phyla (Table S1). To minimize data variation and skewing, we excluded: 1) chordates, which were highly mobile and often moved away while collecting seaweed tissues; 2) copepods, which were relatively abundant on *Desmarestia* spp. throughout the survey; and 3) juvenile crab *Hyas* sp. and nemertean *Tetrastemma* sp., which were rare, on average between zero and one individual per seaweed in any month. For practical reasons, fish (unidentified species) and gastropod (*Lacuna vincta*) egg masses were considered distinct epifaunal entities as opposed to real taxa. Masses (one count per mass) and the 35 remaining taxa were included in the following nMDS, ANOSIM, and SIMPER analyses.

**Figure 4:**
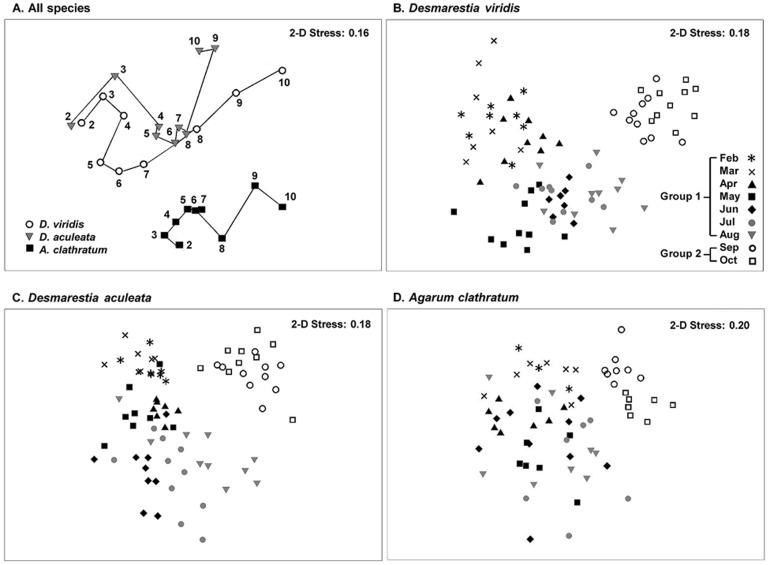
Non-metric multidimensional scaling (nMDS) plots of Bray-Curtis similarities of *Desmarestia viridis*, *Desmarestia aculeata*, and *Agarum clathratum* based on associated epifauna (4^th^-root transformed density, individuals g^−1^ of seaweed) from 18 February to 9 October, 2011 at Keys Point. (A) Each data point is the average of samples for a given month [n = 7 to 10, except 3 for *A. clathratum* in February, for a total n = 221]. The trajectory of change for each seaweed is shown by solid lines connecting consecutive months. Numbers next to symbols indicate sampling month: February (2), March (3), April (4), May (5), June (6), July (7), August (8), September (9), and October (10). (B, C, D) Each data point is one sample within a given month (n =  80 [*D. viridis*], 77 [*D. aculeata*], and 64 [*A. clathratum*]). Group 1 and Group 2 designate clusters of months used in ANOSIM and SIMPER analyses (see Results).

Inspection of the nMDS plot from all monthly averages of samples suggested little difference in the direction of change of epifaunal assemblages among seaweeds throughout the study (Fig. 4A). There was some overlap among assemblages of the three seaweeds (ANOSIM: R = 0.411, p = 0.001) that appeared more pronounced between the two Desmarestiales than between *A. clathratum* and the two Desmarestiales (Fig. 4A). We also noted a marked difference in assemblages of the three seaweeds taken together between the first seven (February to August) and last two (September and October) months (ANOSIM: R = 0.704, p = 0.001; Fig. 4A). The sudden shift in assemblages from August to September coincided well with the end of a rapid (∼2 weeks) increase in mean daily sea temperature, from ∼6°C to ∼12°C, and accelerating release of acid to the environment by, and decay of, *D. viridis* (Fig. 1) [35], [36]. Differences in assemblages between the first seven (Group 1) and last two (Group 2) months were highest in *D. viridis* (ANOSIM: R = 0.736, p = 0.001), followed by *D. aculeata* (ANOSIM: R = 0.629, p = 0.001) and *A. clathratum* (ANOSIM: R = 0.508, p = 0.001) (Fig. 4 B-D). Assemblages in each seaweed exhibited higher similarity percentages in Group 2 than Group 1, ranging from 62% (*D. aculeata*) to 68% (*A. clathratum*), and from 44% (*D. viridis*) to 48% (*A. clathratum*), respectively.

SIMPER analysis of data from February to October (entire survey) showed that the snail *Lacuna vincta* contributed the most to similarities in epifaunal assemblages in the three seaweeds, from ∼23% (*D. viridis* and *D. aculeata*) to ∼34% (*A. clathratum*), followed by the caprellid amphipod *Ischyrocerus anguipes* and mussel *Mytilus* sp. (Table 3). Most of the dissimilarity between *D. viridis* and *D. aculeata* was caused by *L. vincta*, *I. anguipes*, the snail *Margarites helicinus*, and gammarid amphipod *Stenothoe brevicornis*, which were all more abundant (up to two orders of magnitude) on *D. viridis* (Table 3). Patterns of dissimilarity between *D. viridis* and *A. clathratum* were comparable, with the exception that *Mytilus* sp., fish egg masses, and the amphipods *Pontogenia inermis* and *Calliopius laeviusculus* were more abundant on *D. viridis* (Table 3). The polychaete *Spirorbis borealis* and bryozoan *Lichenopora* sp. were generally more abundant on *A. clathratum* than on the two Desmarestiales, thus also contributing to the majority of the differences in epifaunal assemblages among seaweeds. SIMPER analysis of data in September and October (when seaweeds formed distinct clusters) identified *L. vincta* as the numerically dominant species in *D. viridis*, *Mytilus* sp. in *D. aculeata*, and *S. borealis* and *Lichenopora* sp. in *A. clathratum*.

**Table 3:** Epifauna accounting for ≥5% of the similarity within each of the three seaweed species (diagonal) and for ≥5% of the dissimilarity between two seaweeds as determined by SIMPER analysis of data from February to October, 2011.

	Desmarestia viridis		Desmarestia aculeata		Agarum clathratum	
**Desmarestia viridis**	Lacuna vincta	(23.6)				
	Ischyrocerus anguipes	(23.3)				
	Pontogeneia inermis	(7.1)				
	Mytilus sp.	(6.5)				
	Calliopius laeviusculus	(6.1)				
	Fish egg masses	(5.5)				
**Desmarestia aculeata**	Mytilus sp.	(10.7)	L. vincta	(23.3)		
	L. vincta*	(8.2)	Mytilus sp.	(23.1)		
	I. anguipes*	(7.9)	C. laeviusculus	(11.8)		
	P. inermis	(6.5)	I. anguipes	(8.0)		
	C. laeviusculus	(6.2)	M. helicinus	(5.4)		
	Margarites helicinus*	(5.7)				
	Stenothoe brevicornis*	(5.6)				
**Agarum clathratum**	L. vincta*	(9.0)	Mytilus sp.*	(14.9)	L. vincta	(33.5)
	P. inermis*	(8.2)	C. laeviusculus*	(8.1)	I. anguipes	(23.8)
	I. anguipes*	(7.4)	I. anguipes*	(6.4)	S. borealis	(18.5)
	C. laeviusculus*	(7.0)	S. borealis	(6.2)	Mytilus sp.	(7.3)
	Mytilus sp.*	(6.8)	Lichenopora sp.	(5.2)	Lichenopora sp.	(6.8)
	M. helicinus*	(5.8)	M. helicinus*	(5.1)	M. helicinus	(6.2)
	S. brevicornis*	(5.6)	L. vincta*	(5.1)		
	Fish egg masses*	(5.4)				
	Spirorbis borealis	(5.1)				

For each grouping, epifaunal taxa are listed in order of decreasing percentage contribution (bracketed values) to similarity or dissimilarity among seaweed. The density of epifauna with an asterisk is higher on the seaweed given at the top of the table.

**Figure 5:**
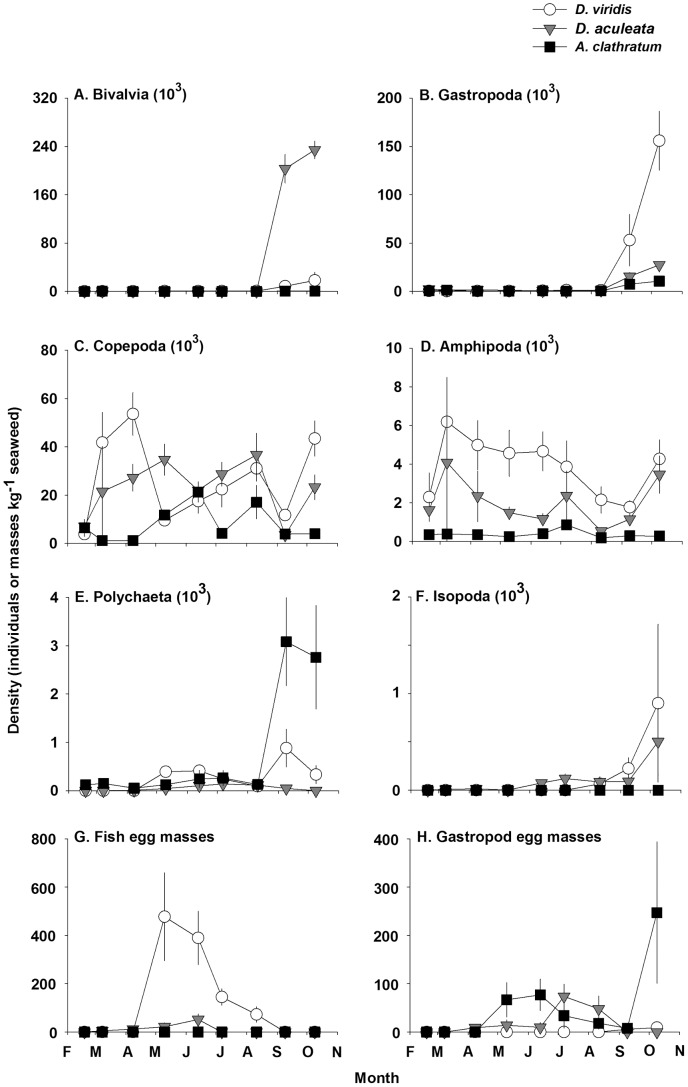
Mean (±SE) density (note the change in scale) of individuals in the six numerically dominant invertebrate taxa and gastropod (*Lacuna vincta*) and fish (unknown species) egg masses associated with *Desmarestia viridis*, *Desmarestia aculeata*, and *Agarum clathratum* from 18 February to 9 October, 2011 at Keys Point (n = 7 to 10 for each data point, except for *A*. *clathratum* in February where n = 3). Bivalvia: *Hiatella arctica, Modiolus modiolus,* and *Mytilus* sp.; Gastropoda: *Dendronotus frondosus, Lacuna vincta,* and *Margarites helicinus*; Copepoda: unidentified species in the Order Harpacticoida; Amphipoda: *Ampithoe rubricata, Calliopius laeviusculus, Caprella linearis, Caprella septentrionalis, Gammarellus angulosus, Gammarus oceanicus, Gammarus setosus, Ischyrocerus anguipes, Leptocheirus pinguis, Pontogeneia inermis,* and *Stenothoe brevicornis*; Polychaeta: *Alitta virens, Autolytinae* sp., *Bylgides sarsi, Lepidonotus squamatus, Nereis pelagica, Phyllodoce mucosa,* and *Spirorbis borealis*; Isopoda: *Idotea baltica* and *Munna* sp.

The density of bivalves, gastropods, copepods, polychaetes, and egg masses (gastropods and fish) differed among seaweeds over time, as shown by the significant interaction between the factors Seaweed and Month (two-way ANOVAs, Table 4). Although copepods were generally more abundant (up to one order of magnitude in most months) than amphipods, these were the only two common taxa from February to October on *D. viridis*, and to a lesser extent on *D. aculeata* (they were largely absent from *A. clathratum*) (Fig. 5). There was a remarkable increase from virtual absence prior to September, to >150 000 gastropod and >230 000 bivalve recruits kg^−1^ of *D. viridis* and *D. aculeata* in October, respectively. Likewise, polychaetes remained low on the two Desmarestiales throughout the survey but increased by three orders of magnitude on *A. clathratum* from August to September (Fig. 5). Isopod density also increased significantly in the last two months regardless of seaweed (Table 4, Fig. 5). Gastropod eggs were consistently uncommon on *D. viridis*. They were predominantly deposited on *A. clathratum* in May and June, on *D. aculeata* in July and August, and again on *A. clathratum* in October, when their density was twice higher than in any other month (Fig. 5). *Desmarestia viridis* was the preferred seaweed for deposition of eggs by fish; egg masses increased by two orders of magnitude from April to May, followed by a steady monthly decline to near absence in September (Fig. 5).

**Table 4:** Summary of two-way ANOVAs (applied to raw data) examining the effect of Seaweed (*Desmarestia viridis*, *Desmarestia aculeata*, and *Agarum clathratum*) and Month (each of nine sampling months: February to October, 2011) on the density of individuals in the six numerically dominant invertebrate taxa, and gastropod (*Lacuna vincta*) and fish (unknown species) egg masses at Keys Point (see caption of Fig. 5 for species in each taxa).

Taxa	Source of variation	*df*	MS	*F*-value	*p*
**Bivalvia**	Seaweed	2	231405	1.70	0.21
	Month	8	179180	1.11	0.41
	Seaweed × Month	16	164396	4.69	<0.01
	Error	194	35018		
	Total	220			
**Gastropoda**	Seaweed	2	153513	1.07	0.37
	Month	8	196991	1.17	0.37
	Seaweed × Month	16	170934	3.67	<0.01
	Error	194	46623		
	Total	220			
**Copepoda**	Seaweed	2	8302.59	19.40	<0.01
	Month	8	2059.23	4.81	<0.01
	Seaweed × Month	16	2115.64	4.94	<0.01
	Error	194	427.92		
	Total	220			
**Amphipoda**	Seaweed	2	263.85	20.35	<0.01
	Month	8	36.92	2.85	<0.01
	Seaweed × Month	16	13.49	1.04	0.42
	Error	194	12.97		
	Total	220			
**Polychaeta**	Seaweed	2	6.33	1.79	0.19
	Month	8	5.82	1.42	0.26
	Seaweed × Month	16	4.17	3.36	<0.01
	Error	194	1.24		
	Total	220			
**Isopoda**	Seaweed	2	0.28	1.01	0.38
	Month	8	0.68	2.66	0.043
	Seaweed × Month	16	0.25	0.71	0.79
	Error	194	0.36		
	Total	220			
**Fish egg masses**	Seaweed	2	0.29	3.51	0.052
	Month	8	0.11	1.18	0.37
	Seaweed × Month	16	0.098	4.16	<0.01
	Error	194	0.024		
	Total	220			
**Gastropod egg masses**	Seaweed	2	0.032	1.62	0.22
	Month	8	0.017	0.81	0.61
	Seaweed × Month	16	0.022	1.75	0.040
	Error	194	0.012		
	Total	220			

**Figure 6:**
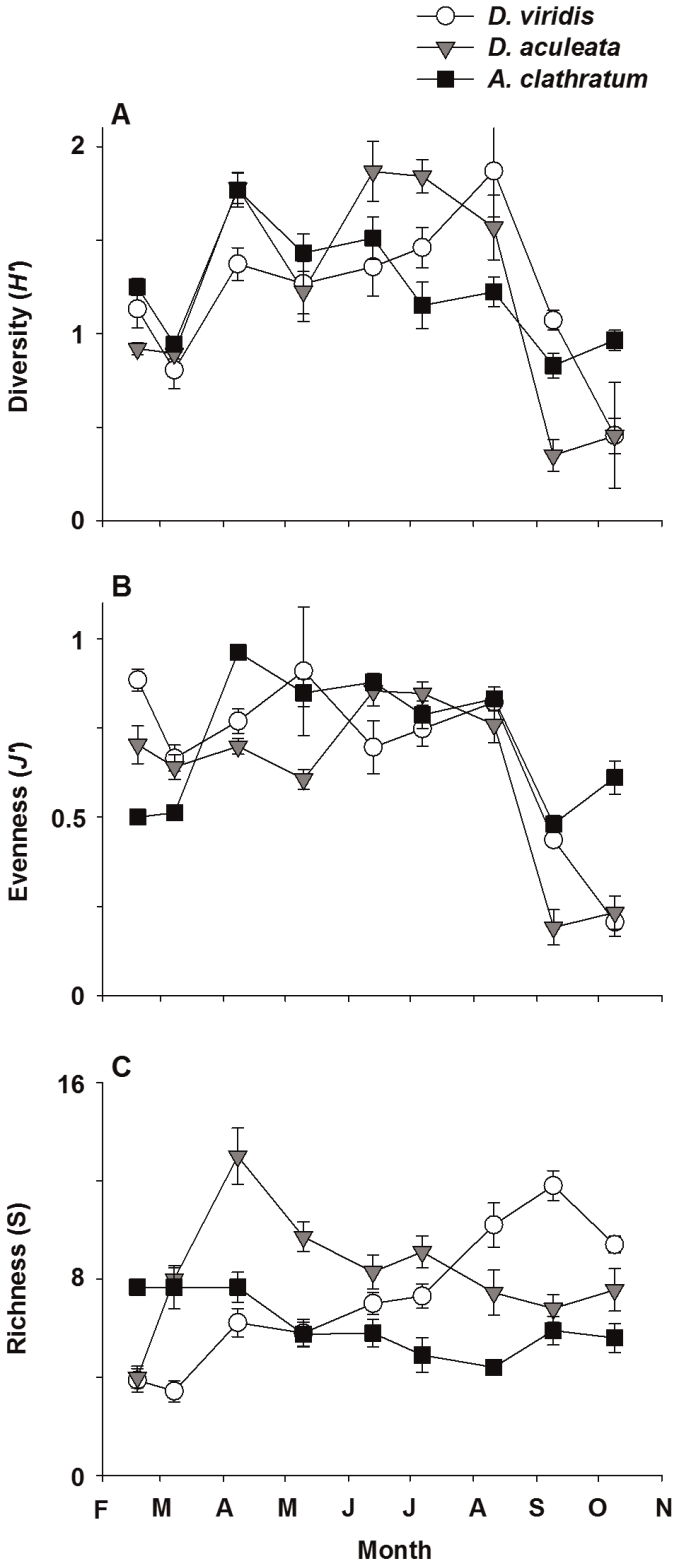
Mean (±SE) Shannon diversity index, *H'* (A), Pielou's evenness index, *J'* (B), and species richness, S (C), of epifauna on *Desmarestia viridis*, *Desmarestia aculeata*, and *Agarum clathratum* from 18 February to 9 October, 2011 at Keys Point (n = 7 to 10 for each data point, except for *A*. *clathratum* in February where n = 3).

The Shannon diversity index differed significantly among seaweeds over time (Table 5). It was generally highest in *A. clathratum* from February to May, in *D. aculeata* in June and July, in *D. viridis* in August and September, and again in *A. clathratum* in October (Fig. 6). There was a two-fold increase in diversity from March to April in all seaweeds, four-fold decrease from August to October in the two Desmarestiales, and two-fold decrease from April to October in *A. clathratum* (Fig. 6). The Pielou's evenness index also differed significantly among seaweeds over time (Table 5). It generally peaked between 0.7 and 0.9 from June to August in all seaweeds (Fig. 6). Evenness in the two Desmarestiales decreased rapidly, by at least three times, in the last two months, whereas it decreased only slightly in *A. clathratum* (Fig. 6). Declines in diversity and evenness from August to October were largely caused by marked increases in recruits of gastropods (*L*. *vincta* and *Margarites helicinus*) in *D. viridis*, and bivalves (*Mytilus* sp. and *Hiatella arctica*) in *D. aculeata*. Species richness also differed significantly among seaweeds over time (Table 5). It generally increased in *D. viridis* from ∼4 to 12 species from March to September (LS means, p<0.001) and decreased in *D. aculeata* from ∼13 to 7 species from April to September (LS means, p<0.001) (Fig. 6). Richness was less variable throughout in *A. clathratum*, ranging from ∼5 (August) to 8 (February to April) species (Fig. 6).

**Table 5:** Summary of two-way ANOVAs (applied to raw data) examining the effect of Seaweed (*Desmarestia viridis*, *Desmarestia aculeata*, and *Agarum clathratum*) and Month (each of nine sampling months: February to October, 2011) on the Shannon diversity index (*H'*), Pielou's evenness index (*J'*), and species richness (S), of seaweed epifauna at Keys Point.

Source of variation	*df*	MS	*F*-value	*p*
Diversity (*H'*)				
Seaweed	2	0.016	0.15	0.85
Month	8	3.85	37.83	<0.01
Seaweed × Month	16	0.70	6.90	<0.01
Error	194	0.10		
Total	220			
Evenness (*J'*)				
Seaweed	2	0.15	10.78	<0.01
Month	8	0.99	71.31	<0.01
Seaweed × Month	16	0.13	9.60	<0.01
Error	194	0.014		
Total	220			
Species richness (S)				
Seaweed	2	62.20	18.58	<0.01
Month	8	19.15	5.72	<0.01
Seaweed × Month	16	41.16	12.29	<0.01
Error	194	3.35		
Total	220			

### Vulnerability to grazing

**Figure 7:**
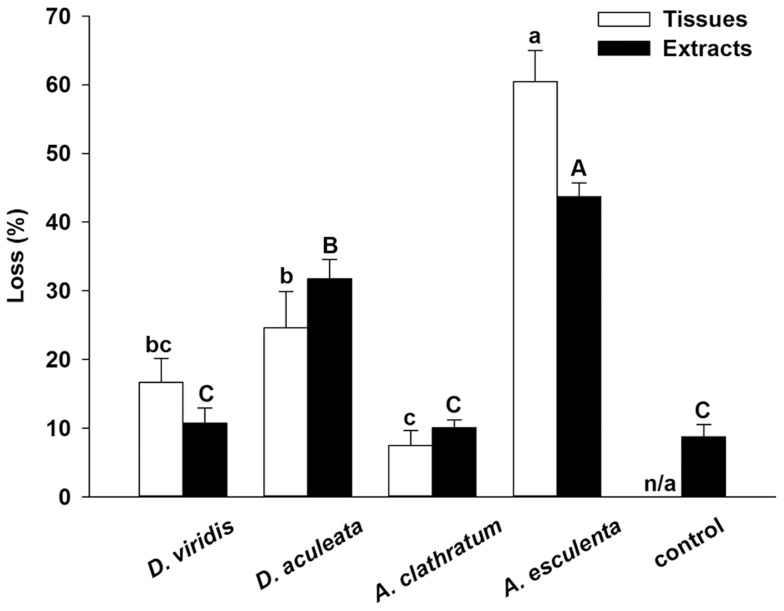
Loss in mean (+SE) wet weight as a percentage of initial wet weight of tissues (Experiment 1) and agar-embedded extracts (Experiment 2) of *Desmarestia viridis*, *Desmarestia aculeata, Agarum clathratum*, and *Alaria esculenta* sporophytes exposed 48 h to grazing by 10 green sea urchins, *Strongylocentrotus droebachiensis*. Bars not sharing the same letter are different (LS means tests, p<0.05; n = 15 [Experiment 1] and 20 [Experiment 2]) (see “Materials and methods” for a description of each experiment and nature of the procedural control in Experiment 2).

Analysis of data from Experiment 1 showed that the loss of sporophyte tissues to urchin grazing was similar between *Desmarestia viridis* and *Desmarestia aculeata* and between *D. viridis* and *Agarum clathratum*, and at least 59% less in any of these three seaweeds compared to *Alaria esculenta* (one-way ANOVA [generalized linear model]: Factor  =  Seaweed, χ^2^ = 12.01, p = 0.0074, Fig. 7). Analysis of data from Experiment 2 with agar-embedded extracts of grinded sporophyte tissues showed a slightly different outcome whereby grazing on *D. viridis* was significantly lower than on *D. aculeata* but similar to *A. clathratum* (one-way ANOVA [generalized linear model]: Factor  =  Seaweed, χ^2^ = 11.64, p = 0.020, Fig. 7). As in Experiment 1, grazing was highest on *A. esculenta*, followed by *D. aculeata* with a difference of 27% between the two (χ^2^ = 10.81, p = 0.001). The loss of agar-embedded extracts of *D. viridis* and *A. clathratum* was similar to that of the procedural control with no seaweed extracts (Fig. 7), supporting the notion that the chemical makeup of both seaweeds was not particularly attractive to urchins.

## Discussion

This study uncovers biological, ecological, and functional aspects of the annual, brown seaweed *Desmarestia viridis* that advocate fundamental changes to the study of community shifts in shallow, rocky ecosystems in eastern Canada.

### The need to consider a transient community state: “*Desmarestia* bed”

We showed that *D. viridis* can form a transient canopy from March to October over bare or coralline seaweed-encrusted rocky substratum within the 2-to-8 m depth range in Bay Bulls, Newfoundland. The canopy covered, on average, ∼25% of the seabed in 2011, and up to ∼90% in 2012 and 2013. Interestingly, this depth range is also where destructive grazing of kelp beds by urchin fronts typically occur in eastern Canada [28], [30]. The initiation of the *D. viridis* canopy in March and April was when significant wave height and sea temperature were nearing annual maxima and minima, respectively, and urchin displacement and grazing were greatly limited. During the same period, *D. viridis* sporophytes exhibited relatively high mortality (up to 40% bi-weekly) and growth (∼4 to 6% daily increase in length), and the intracellular acidity also increased rapidly [35], [36]. These observations suggest that recruits depend on the severity of the physical environment to escape grazing while they invest resources in the production of tissues and sulfuric acid. Yet, fine-scale variation in the physical and biological environments could affect the establishment of *D. viridis*, as implied by between- and within-site differences in *D. viridis* cover, and inverse relationships between *D. viridis* cover and urchin density at 3 and 4 m at BCC, and at 3, 4, and 8 m at KP.

In a concurrent study, we found that significant wave height was generally higher at KP than at BCC from March to early July and from mid-August to October (no difference between sites from early July to mid-August) [35]. These patterns and the generally decreasing water flows from 2 to 8 m that we perceived during our dives at both sites, may partly explain the observed site- and depth-specific differences in *D. viridis* and urchin abundances. This suggestion is corroborated by the experimental demonstration that moderate, wave-induced sweeping motion of *D. viridis* sporophytes provides mechanical protection against urchin grazing up to a threshold of 136 to 194 urchins m^−2^ [40]. Accordingly, we think that 1) high wave action at 2 m at BCC and KP largely prevented urchins from moving and grazing fast-sweeping *D. viridis* sporophytes; 2) moderate wave action at 3 and 4 m at BCC, and from 3 to 8 m at KP, provided *D. viridis* with efficient mechanical protection against urchin grazing at relatively low urchin densities; and 3) low wave action at 8 m at BCC did not induce sufficient sweeping in *D. viridis*, which was readily grazed by urchins, even at low densities.

As noted above, we witnessed the establishment of a virtually continuous *D. viridis* canopy in 2012 and 2013 throughout much of the 3-to-8 m depth range at our two study sites. This phenomenon certainly is not unique to our study sites. Canopies of *D. viridis* frequently develop in urchin barrens, often right below the lower edge of kelp beds, throughout the extensive (17542 km) coast of Newfoundland and Labrador ([48], [57], [58], [59], P. Gagnon personal observations). This is also the case in the northern Gulf of St. Lawrence [11], [28], [33], [42] and Nova Scotia [30], [31]. In these regions, the cover of *D. viridis* often momentarily exceeds 70% on vertical surfaces (e.g. boulders and rock walls), while nearing 100% on horizontal surfaces (see aforementioned references). Using data from 41 stations at 26 sites along the coasts of northeastern Newfoundland, southern Labrador, and eastern Québec, Adey and Hayek [57] show that *D. viridis* can form up to 39% of the total seaweed biomass within the 2.5-to-10 m depth range. They also show that *D. viridis* and the kelp *Agarum clathratum* overwhelmingly dominate the lower half to two-thirds of the subtidal macrophyte zone in the Newfoundland-Labrador-Québec region [57], which Gagnon et al. [34] further corroborate.

Therefore, we propose that *D. viridis* recruitment, especially in years of higher productivity, results in the creation of an ephemeral, or transient, community state termed “*Desmarestia* bed”, adding to the kelp bed and urchin barren states in eastern Canada. Several studies in eastern Canada and the Aleutian (northern Pacific) and Svalbard (Arctic Ocean) archipelagos suggest that *D. viridis* sporophytes function as “giant sweepers” that retard or prevent the formation of urchin fronts at the lower edge of kelp beds [40], [43], [48], [60]. In eastern Canada, the predictable annual outbreak and die-off of *D. viridis* sporophytes could then represent a cyclical, natural disturbance that disrupts urchin-kelp interactions, ultimately allowing kelp beds to re-establish over the barrens. Longer-term studies of the relationships between the physical environment, *D. viridis* and urchin abundances, and the frequency and extent to which kelp beds re-establish over barrens colonized or not by *Desmarestia* beds, are needed to determine the importance of *D. viridis* to ecosystem resilience [61], [62].

### The need to elevate *D. viridis* to the rank of foundation species

We showed that the quick development of *Desmarestia* beds in urchin barrens creates biological structure for major recruitment pulses in characteristic invertebrate and fish assemblages. Most recruitment pulses measured at KP (and observed at BCC), including the herbivorous snail *Lacuna vincta* from quasi-absent to >150 000 recruits kg^−1^ *D. viridis*, were restricted to only a few weeks in August and September. At this time mean sea temperature fluctuated around the lethal 12°C for *D. viridis*: the sulfuric acid was released from *D. viridis* to the environment, the sporophytes entered the senescence phase, and urchins began to massively graze *D. viridis* ([35], [36], this study). Such annual, synchronous decay in *D. viridis* also occur throughout the rest of Newfoundland and Labrador, the northern Gulf of St. Lawrence, and Nova Scotia, ([11], [30], [57], P. Gagnon unpublished data). It suggests complex, environmentally- (largely temperature) driven cascades. In this case, a self-defended, fast-growing seaweed suddenly turns into a highly vulnerable prey for numerically dominant benthic (e.g. urchin) and epifaunal grazers (e.g. *L. vincta*, see below). We found that fish egg masses occurred almost exclusively on *D. viridis*, whereas *L. vincta* and other invertebrates deposited eggs or recruited almost exclusively on the two longer-lived, perennial seaweeds *Desmarestia aculeata* and *A. clathratum*. These patterns further suggest ontogenetic partitioning in the use of habitat-forming species among invertebrates.

By forming large aggregations relative to the size of the organisms that they facilitate, marine foundation species markedly increase environmental heterogeneity, often transforming a two-dimensional, featureless landscape into a complex, three-dimensional structure [18]. Our study supports the notion that *D. viridis*, and perhaps *D. aculeata* and *A. clathratum*, facilitate recruitment of distinct groups of invertebrates and fish, and hence function as foundation species in urchin barrens. Specifically, we showed that epifaunal assemblages in the three seaweeds were 1) composed almost exclusively [∼75%] of recruits and juveniles; 2) more stable in *A. clathratum* than the two Desmarestiales from February to August; 3) more similar [diversity and evenness] from February to August between the two Desmarestiales than between any of the Desmarestiales and *A. clathratum*; and 4) markedly different in September and October than in the previous eight months in each seaweed. The rapid decline in diversity at the end of the season appeared to be mainly due to increasing dominance within communities, while richness remained relatively constant. In a study of invertebrate assemblages associated with seaweed canopies in the northern Gulf of St. Lawrence, Bégin et al. [33] found only trace abundance of *L. vincta* and the snail *Margarites helicinus* on fronds of *D. viridis*. They also reported a higher invertebrate diversity on *A. clathratum* than *D. viridis* [33]. By sampling a broader range of invertebrate sizes (250+ μm versus 1+ mm) over a longer period (nine months versus two months) than Bégin et al. [33], the present study draws opposite conclusions. It shows that *Desmarestia viridis* sporophytes can actually be heavily colonized by *L. vincta* and *M. helicinus*, in addition to many other invertebrates common to both studies. Sporophytes can also support a generally higher, albeit less even, epifaunal diversity and species richness than *A. clathratum* in the last few months of existence of *D. viridis*. Therefore, our findings also underscore the importance of studying organisms together with cross-scale environmental variability to better understand the factors and processes that shape marine communities.

We demonstrated experimentally that urchin grazing on non-sweeping sporophyte tissues (Experiment 1) and agar-embedded extracts of grinded sporophyte tissues (Experiment 2) is equally lower in *D. viridis* and *A. clathratum* than on *D. aculeata* and the preferred kelp *Alaria esculenta*. That the two experiments provided fairly similar results with regards to the ranking of seaweeds, suggests that in the absence of waves and currents vulnerability to grazing in *D. viridis* and *A. clathratum*, and to a lesser extent in *D. aculeata*, depends primarily on the chemical makeup. Morphological or structural differences (e.g. tissue toughness) if any, were not sufficient to cause the observed differences in grazing. These experiments were not designed to identify the chemical compounds at the origin of the observed differences in grazing. Nevertheless, results of the two experiments taken together indicate that the chemical makeup of *D. viridis* and *A. clathratum* helps retard urchin grazing compared to *D. aculeata* and *A. esculenta*. They corroborate other studies that suggest that the sulfuric acid in *D. viridis*, and phenolics in *A. clathratum*, act as chemical deterrents to grazing [11], [40], [43]–[45]. Further research is needed to determine the relative importance of the chemical makeup and wave-induced sweeping motion of fronds to the survival of these seaweeds in urchin barrens.

### A novel empirical model of ecological interactions for *D. viridis*

Several studies have helped elevate the ecological significance of unusual morphology and acid (H_2_SO_4_) production of *D. viridis* in eastern Canada. The wave-induced sweeping motion and chemical makeup of *D. viridis* sporophytes provide density-dependent protection against grazing [40]. This natural protection ultimately enables the species to form extensive canopies in urchin barrens ([28], [30], [57], this study) that enhance the recruitment of other seaweeds [11]. The constitutive and irreversible accumulation of acid as sporophytes grow from recruits to adults inevitably culminates into dramatic mass releases of acid and die offs, when mean sea temperature rises above 12°C (the species is intolerant to temperature above ∼12°C) [35], [36]. These and the present studies provide the empirical evidence needed to propose a novel model of ecological interactions for the species that can serve as a foundation for future studies of community shifts in rocky subtidal ecosystems in eastern Canada.

**Figure 8:**
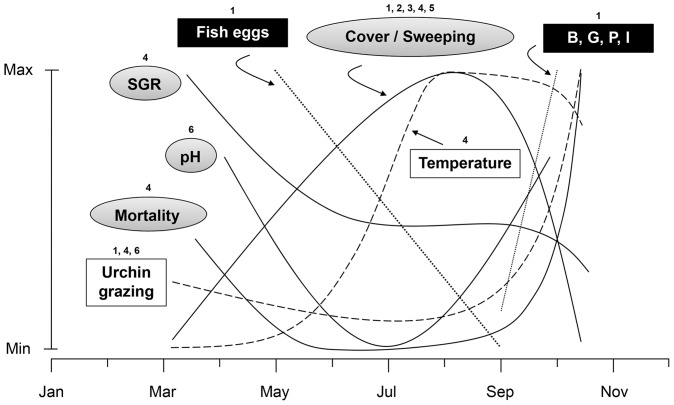
Empirical model of ecological interactions in sporophytes of the annual, acidic (H_2_SO_4_), brown seaweed *Desmarestia viridis* in urchin (*Strongylocentrotus droebachiensis*) barrens in eastern Canada throughout an entire growth season (March to October). The dominant environmental controls (temperature and urchin grazing) are shown as open rectangles and dashed lines, *D. viridis* traits (cover/sweeping, specific growth rate [SGR], pH, and mortality) as gray ellipses and solid lines, and epifauna (fish eggs, bivalves [B], herbivorous gastropods [G], and predatory polychaetes [P] and isopods [I]) as solid rectangles and dotted lines. Numbers above rectangles and ellipses are the sources of information for temporal variation: 1 [present study]; 2 [28]; 3 [30]; 4 [35]; 5 [57]; 6 [36] (see “Discussion” for a detailed description of the model).

According to this model (Fig. 8), small (∼10 to 15 cm in length) *D. viridis* recruits exhibit highest specific growth rates (SGR) in early March, when the cover and amplitude of the wave-induced sweeping motion are lowest, and mortality and grazing by urchins are moderate. As recruits grow to adult-size (∼50 to 60 cm) sporophytes in July, SGR and intracellular pH decrease (the latter being indicative of sulfuric acid production and accumulation) at a decelerating rate. Mortality decreases as a result of increasing cover and sweeping, which further reduces urchin grazing. The rapid increase in mean sea temperature in June and July to above 10°C marks the onset of mass release of acid to the environment (as shown by increasing pH), and senescence (as shown by sudden declines in cover and SGR) until all sporophytes disappear in October. The sudden increase in urchin grazing on decaying sporophytes in August and September precipitates mortality and decline in cover. Throughout its existence as a sporophyte, *D. viridis* provides a suitable surface for deposition of eggs by fish, as well as to distinct assemblages of mobile epifauna throughout the senescence phase. These patterns of variation mainly reflect conditions at the two study sites in the present study. Patterns may change slightly with location because of the importance of the thermal environment to the biology of *D. viridis* (see above) and latitudinal variation in sea temperature in eastern Canada [35], [63]–[65].

### Summary and future research directions

The present and related studies demonstrate the ability of *Desmarestia viridis* to form relatively large (10s to 100s of m^2^) canopies, namely *Desmarestia* beds, in shallow rocky ecosystems in eastern Canada. These canopies, together with those of two co-occurring, longer-lived seaweeds in urchin barrens, *Desmarestia aculeata* and *Agarum clathratum*, can host high abundances of recruits, juveniles, and adults in at least 41 invertebrate and fish taxa, indicating a strong foundational potential. The annual resurgence and high spatial and temporal predictability of *Desmarestia* beds and associated epifauna represent a third community state adding to the much more studied kelp bed and urchin barrens states. In light of our findings and the patterns they suggest, we propose that canopy-forming seaweeds in so-called urchin barrens play an underappreciated role in the overall ecosystem dynamics. In particular, we think that the traditional view that shallow rocky subtidal ecosystems in eastern Canada alternate between two community states, kelp beds and urchin barrens [28], [29], [30], is, at best, incomplete. This view should be broadened by a more inclusive examination of the contribution to ecosystem resilience of *D. viridis* and other canopy-forming seaweeds in urchin barrens. For example, the prospect that *Desmarestia* beds disrupt cyclical alternations between the kelp bed and urchin barrens states, as suggested for *A. clathratum* in the northern Gulf of St. Lawrence [28], [34], must be investigated through long-term, manipulative experiments, and multiyear mensurative studies. Doing so would also help determine whether *Desmarestia* beds form a stable alternative basin of attraction [66], [67] for epifaunal assemblages, or a successional stage in the trajectory towards the kelp bed. Finally, our study advocates the need to regard certain canopy-forming seaweeds as structuring forces interfering with top-down processes, rather than simple prey for keystone grazers. It therefore adds to recent calls for critical reassessments of the generality of phase-shifts and their mechanisms in iconic marine ecosystems, including kelp beds [68], [69].

## Supporting Information

Table S1 Mean density (individuals [or egg masses] kg^−1^ seaweed) of invertebrates and fish on *Desmarestia viridis*, *Desmarestia aculeata*, and *Agarum clathratum* sporophytes sampled monthly from 18 February to 9 October, 2011. Standard error is given below each mean in *italics*.
(DOCX)
