# Supplementary material for: Canopy-Forming Seaweeds in Urchin-Dominated Systems in Eastern Canada: Structuring Forces or Simple Prey for Keystone Grazers?
Source: PLoS One. 2014 May 23;9(5):e98204. doi: 10.1371/journal.pone.0098204 (PMC4032334; doi:10.1371/journal.pone.0098204)
Supplement: Table S1 — Mean density (individuals [or egg masses] kg−1 seaweed) of invertebrates and fish on Desmarestia viridis, Desmarestia aculeata, and Agarum clathratum sporophytes sampled monthly from 18 February to 9 October, 2011. Standard error is given below each mean in italics. (DOCX) [file pone.0098204.s001.docx]

***Desmarestia viridis***

|  |  |  |  |  |  |  |  |  |  |
| --- | --- | --- | --- | --- | --- | --- | --- | --- | --- |
| **Taxon** | **Feb** | **Mar** | **Apr** | **May** | **Jun** | **Jul** | **Aug** | **Sep** | **Oct** |
| **Annelida** |  |  |  |  |  |  |  |  |  |
| **Polychaeta** |  |  |  |  |  |  |  |  |  |
| *Alitta virens* M Sars | 0 | 0 | 0 | 0 | 0 | 0 | 0 | 0 | 0 |
|  | *0.0* | *0.0* | *0.0* | *0.0* | *0.0* | *0.0* | *0.0* | *0.0* | *0.0* |
| *Autolytinae sp.* | 0 | 0 | 0 | 189.9 | 211.2 | 140.7 | 19.8 | 44.5 | 0 |
|  | *0.0* | *0.0* | *0.0* | *22.1* | *34.5* | *65.4* | *11.0* | *15.5* | *0.0* |
| *Bylgides sarsi* Kinberg in Malmgren | 0 | 0 | 0 | 87.4 | 131.7 | 90.0 | 69.6 | 15.6 | 0 |
|  | *0.0* | *0.0* | *0.0* | *22.8* | *16.8* | *45.1* | *23.7* | *3.4* | *0.0* |
| *Lepidonotus squamatus* Linnaeus | 0 | 0 | 0 | 0 | 0 | 0 | 0 | 0 | 0 |
|  | *0.0* | *0.0* | *0.0* | *0.0* | *0.0* | *0.0* | *0.0* | *0.0* | *0.0* |
| *Nereis pelagica* Linnaeus | 0 | 0 | 0 | 0 | 0 | 0 | 0 | 0 | 0 |
|  | *0.0* | *0.0* | *0.0* | *0.0* | *0.0* | *0.0* | *0.0* | *0.0* | *0.0* |
| *Phyllodoce mucosa* Örsted | 0 | 0 | 0 | 121.4 | 69.5 | 0 | 0 | 0 | 0 |
|  | *0.0* | *0.0* | *0.0* | *22.4* | *13.4* | *0.0* | *0.0* | *0.0* | *0.0* |
| *Spirorbis borealis* Daudin | 0 | 0 | 0 | 0 | 0 | 0 | 0 | 821.8 | 332.8 |
|  | *0.0* | *0.0* | *0.0* | *0.0* | *0.0* | *0.0* | *0.0* | *119.2* | *56.7* |
| **Arthropoda** |  |  |  |  |  |  |  |  |  |
| **Arachnida** |  |  |  |  |  |  |  |  |  |
| Halacaridae | 0 | 132.3 | 16.7 | 18.0 | 17.8 | 23.0 | 138.4 | 69.8 | 0 |
|  | *0.0* | *5.6* | *2.9* | *3.8* | *3.8* | *5.8* | *25.8* | *14.1* | *0.0* |
| **Crustacea** |  |  |  |  |  |  |  |  |  |
| *Ampithoe rubricata* Montagu | 0 | 0 | 162.1 | 81.1 | 0.0 | 0.0 | 39.9 | 13.5 | 95.1 |
|  | *0.0* | *0.0* | *16.7* | *21.3* | *0.0* | *0.0* | *18.4* | *6.4* | *31.1* |
| *Calliopius laeviusculus* Kroyer | 26.4 | 15.0 | 1232.8 | 527.4 | 378.4 | 142.1 | 1102.3 | 122.0 | 314.8 |
|  | *7.7* | *4.5* | *46.9* | *32.1* | *33.5* | *21.0* | *66.7* | *23.9* | *29.4* |
| *Caprella linearis* Linnaeus | 866.5 | 621.0 | 224.9 | 38.9 | 0 | 0 | 0 | 137.8 | 227.7 |
|  | *56.4* | *87.7* | *43.5* | *18.0* | *0.0* | *0.0* | *0.0* | *25.2* | *42.2* |
| *Caprella septentrionalis* Kroyer | 81.6 | 199.5 | 0 | 0 | 0 | 0 | 0 | 10.3 | 0 |
|  | *10.9* | *14.7* | *0.0* | *0.0* | *0.0* | *0.0* | *0.0* | *3.9* | *0.0* |
| Copepoda* | 4185.5 | 41644.1 | 53571.4 | 9945.5 | 17533.0 | 21435.6 | 31042.2 | 11772.8 | 45469.4 |
|  | *1087.7* | *2184.8* | *2544.4* | *2138.9* | *4887.8* | *8997.2* | *9925.5* | *3589.4* | *9844.8* |
| *Gammarellus angulosus* Rathke | 0 | 0 | 33.1 | 0 | 0 | 0 | 3.8 | 0 | 0 |
|  | *0.0* | *0.0* | *12.0* | *0.0* | *0.0* | *0.0* | *2.6* | *0.0* | *0.0* |
| *Gammarus oceanicus* Segerstråle | 107.5 | 522.9 | 34.7 | 5.0 | 0 | 0 | 17.4 | 0 | 49.5 |
|  | *33.3* | *48.1* | *16.5* | *3.5* | *0.0* | *0.0* | *9.1* | *0.0* | *22.0* |
| *Gammarus setosus* Dementieva | 0 | 0 | 0 | 0 | 0 | 0 | 0 | 3.2 | 0.0 |
|  | *0.0* | *0.0* | *0.0* | *0.0* | *0.0* | *0.0* | *0.0* | *2.0* | *0.0* |
| *Hyas sp.** | 0 | 0 | 0 | 0 | 0 | 0 | 0 | 0 | 0 |
|  | *0.0* | *0.0* | *0.0* | *0.0* | *0.0* | *0.0* | *0.0* | *0.0* | *0.0* |
| *Idotea baltica* Pallas | 0 | 0 | 0 | 0 | 0 | 0 | 40.2 | 188.5 | 875.5 |
|  | *0.0* | *0.0* | *0.0* | *0.0* | *0.0* | *0.0* | *9.8* | *29.3* | *69.1* |
| *Ischyrocerus anguipes* Kroyer | 1165.3 | 3701.5 | 2023.2 | 3573.4 | 3264.0 | 2932.4 | 316.3 | 454.2 | 133.6 |
|  | *169.5* | *284.1* | *155.2* | *281.0* | *139.9* | *157.3* | *134.5* | *99.7* | *87.0* |
| *Leptocheirus pinguis* Stimpson | 0 | 0 | 0 | 0 | 316.5 | 370.6 | 251.4 | 182.8 | 118.1 |
|  | *0.0* | *0.0* | *0.0* | *0.0* | *68.5* | *46.0* | *55.5* | *31.7* | *28.9* |
| *Munna sp.* | 0 | 0 | 0 | 0 | 0 | 0 | 31.8 | 51.7 | 65.8 |
|  | *0.0* | *0.0* | *0.0* | *0.0* | *0.0* | *0.0* | *8.8* | *11.7* | *15.9* |
| *Pontogeneia inermis* Kroyer | 72.0 | 547.8 | 570.9 | 343.6 | 590.7 | 288.7 | 222.6 | 430.0 | 2143.3 |
|  | *22.4* | *101.8* | *83.6* | *75.8* | *88.0* | *57.4* | *44.0* | *43.8* | *101.4* |
| *Stenothoe brevicornis* G.O. Sars | 0 | 522.6 | 663.7 | 0 | 74.2 | 19.0 | 229.7 | 374.9 | 1318.0 |
|  | *0.0* | *101.5* | *165.2* | *0.0* | *19.7* | *11.5* | *77.3* | *75.1* | *185.2* |
| **Pycnogonida** |  |  |  |  |  |  |  |  |  |
| *Phoxichilidium femoratum* Rathke | 0 | 0 | 0 | 0 | 0 | 0 | 0 | 10.7 | 77.7 |
|  | *0.0* | *0.0* | *0.0* | *0.0* | *0.0* | *0.0* | *0.0* | *4.5* | *12.2* |
| **Chordata** |  |  |  |  |  |  |  |  |  |
| **Actinopterygii** |  |  |  |  |  |  |  |  |  |
| *Cyclopterus lumpus* Linnaeus* | 0 | 0 | 0 | 0 | 0 | 0 | 0 | 0 | 0 |
|  | *0.0* | *0.0* | *0.0* | *0.0* | *0.0* | *0.0* | *0.0* | *0.0* | *0.0* |
| *Liparis inquilinus* Able* | 0 | 0 | 0 | 0 | 0 | 0 | 0 | 0 | 0 |
|  | *0.0* | *0.0* | *0.0* | *0.0* | *0.0* | *0.0* | *0.0* | *0.0* | *0.0* |
| *Pholis gunnellus* Linnaeus* | 0 | 0 | 0 | 0 | 2.9 | 1.8 | 3.2 | 0 | 0 |
|  | *0.0* | *0.0* | *0.0* | *0.0* | *0.2* | *1.9* | *2.8* | *0.0* | *0.0* |
| Egg masses (unknown species) | 0 | 10.0 | 20.1 | 477.4 | 389.5 | 145.0 | 72.5 | 5.3 | 0 |
|  | *0.0* | *3.1* | *11.8* | *79.8* | *66.7* | *68.4* | *30.8* | *2.9* | *0.0* |
| **Cnidaria** |  |  |  |  |  |  |  |  |  |
| **Hydrozoa** |  |  |  |  |  |  |  |  |  |
| *Obelia geniculata* Linnaeus** | 0 | 0 | 0 | 0 | 0 | 0 | 2.8 | 0 | 0 |
|  | *0.0* | *0.0* | *0.0* | *0.0* | *0.0* | *0.0* | *0.4* | *0.0* | *0.0* |
| **Scyphozoa** |  |  |  |  |  |  |  |  |  |
| *Haliclystus salpinx* James-Clark | 0 | 0 | 0 | 0 | 0 | 0 | 39.4 | 20.1 | 167.4 |
|  | *0.0* | *0.0* | *0.0* | *0.0* | *0.0* | *0.0* | *15.2* | *5.2* | *43.1* |
| **Echinodermata** |  |  |  |  |  |  |  |  |  |
| **Asteroidea** |  |  |  |  |  |  |  |  |  |
| *Asterias sp.* | 0 | 0 | 0 | 0 | 0 | 0 | 0 | 37.0 | 58.1 |
|  | *0.0* | *0.0* | *0.0* | *0.0* | *0.0* | *0.0* | *0.0* | *13.0* | *12.4* |
| **Ophiuroidea** |  |  |  |  |  |  |  |  |  |
| *Ophiopholis aculeata* Linnaeus | 0 | 0 | 0 | 0 | 0 | 0 | 0 | 0 | 0 |
|  | *0.0* | *0.0* | *0.0* | *0.0* | *0.0* | *0.0* | *0.0* | *0.0* | *0.0* |
| **Ectoprocta** |  |  |  |  |  |  |  |  |  |
| **Gymnolaemata** |  |  |  |  |  |  |  |  |  |
| *Electra pilosa* Linnaeus** | 0 | 0 | 0 | 0 | 0 | 0 | 0 | 0 | 37.8 |
|  | *0.0* | *0.0* | *0.0* | *0.0* | *0.0* | *0.0* | *0.0* | *0.0* | *14.7* |
| **Stenolaemata** |  |  |  |  |  |  |  |  |  |
| *Lichenopora sp*.** | 0 | 0 | 0 | 0 | 0 | 0 | 0 | 0 | 0 |
|  | *0.0* | *0.0* | *0.0* | *0.0* | *0.0* | *0.0* | *0.0* | *0.0* | *0.0* |
| **Mollusca** |  |  |  |  |  |  |  |  |  |
| **Bivalvia** |  |  |  |  |  |  |  |  |  |
| *Hiatella arctica* Linnaeus | 0 | 0 | 0 | 0 | 0 | 0 | 0 | 11838.7 | 1454.3 |
|  | *0.0* | *0.0* | *0.0* | *0.0* | *0.0* | *0.0* | *0.0* | *1534.1* | *299.0* |
| *Modiolus modiolus* Linnaeus | 0 | 0 | 0 | 187.0 | 101.7 | 155.7 | 492.0 | 0 | 0 |
|  | *0.0* | *0.0* | *0.0* | *45.8* | *33.4* | *21.0* | *85.6* | *0.0* | *0.0* |
| *Mytilus sp.* | 40.5 | 13.0 | 16.7 | 163.7 | 262.1 | 292.7 | 133.6 | 7931.4 | 26539.1 |
|  | *6.8* | *1.5* | *1.6* | *24.5* | *22.7* | *23.9* | *11.0* | *156.5* | *5897.5* |
| **Gastropoda** |  |  |  |  |  |  |  |  |  |
| *Dendronotus frondosus* Ascanius | 0 | 0 | 0 | 0 | 0 | 0 | 31.1 | 145.0 | 261.3 |
|  | *0.0* | *0.0* | *0.0* | *0.0* | *0.0* | *0.0* | *4.8* | *232.8* | *99.6* |
| *Lacuna vincta* Montagu | 481.1 | 164.7 | 344.1 | 308.8 | 696.7 | 1035.5 | 1041.9 | 44885.5 | 154770.4 |
|  | *45.5* | *21.7* | *40.2* | *19.9* | *101.8* | *245.7* | *321.8* | *983.3* | *5994.5* |
| *Margarites helicinus* Phipps | 0 | 0 | 0 | 0 | 0 | 196.6 | 111.5 | 7921.7 | 3986.6 |
|  | *0.0* | *0.0* | *0.0* | *0.0* | *0.0* | *22.8* | *13.4* | *389.5* | *677.0* |
| Egg masses (*L. vincta*) | 0 | 0 | 0 | 0 | 0 | 0 | 0 | 5.5 | 9.1 |
|  | *0.0* | *0.0* | *0.0* | *0.0* | *0.0* | *0.0* | *0.0* | *1.2* | *1.3* |
| **Polyplacophora** |  |  |  |  |  |  |  |  |  |
| *Tonicella marmorea* Fabricius | 0 | 0 | 0 | 0 | 0 | 0 | 0 | 0 | 0 |
|  | *0.0* | *0.0* | *0.0* | *0.0* | *0.0* | *0.0* | *0.0* | *0.0* | *0.0* |
| **Nemertea** |  |  |  |  |  |  |  |  |  |
| **Enopla** |  |  |  |  |  |  |  |  |  |
| *Tetrastemma sp.** | 0 | 5.2 | 19.8 | 0 | 0 | 20.8 | 53.4 | 0 | 0 |
|  | *0.0* | *1.0* | *2.9* | *0.0* | *0.0* | *3.1* | *8.8* | *0.0* | *0.0* |
|  |  |  |  |  |  |  |  |  |  |

***Desmarestia aculeata***

|  |  |  |  |  |  |  |  |  |  |
| --- | --- | --- | --- | --- | --- | --- | --- | --- | --- |
| **Taxon** | **Feb** | **Mar** | **Apr** | **May** | **Jun** | **Jul** | **Aug** | **Sep** | **Oct** |
| **Annelida** |  |  |  |  |  |  |  |  |  |
| **Polychaeta** |  |  |  |  |  |  |  |  |  |
| *Alitta virens* M Sars | 0 | 0 | 0 | 0 | 0 | 0 | 0 | 0 | 0 |
|  | *0.0* | *0.0* | *0.0* | *0.0* | *0.0* | *0.0* | *0.0* | *0.0* | *0.0* |
| *Autolytinae sp.* | 0 | 0 | 0 | 45.5 | 45.2 | 0 | 0 | 0 | 0 |
|  | *0.0* | *0.0* | *0.0* | *12.3* | *14.2* | *0.0* | *0.0* | *0.0* | *0.0* |
| *Bylgides sarsi* Kinberg in Malmgren | 0 | 0 | 0 | 0 | 64.7 | 73.0 | 33.2 | 10.0 | 0 |
|  | *0.0* | *0.0* | *0.0* | *0.0* | *13.2* | *15.7* | *11.0* | *2.5* | *0.0* |
| *Lepidonotus squamatus* Linnaeus | 0 | 0 | 0 | 0 | 0 | 0 | 0 | 0 | 0 |
|  | *0.0* | *0.0* | *0.0* | *0.0* | *0.0* | *0.0* | *0.0* | *0.0* | *0.0* |
| *Nereis pelagica* Linnaeus | 0 | 0 | 0 | 0 | 0 | 0 | 0 | 0 | 0 |
|  | *0.0* | *0.0* | *0.0* | *0.0* | *0.0* | *0.0* | *0.0* | *0.0* | *0.0* |
| *Phyllodoce mucosa* Örsted | 0 | 0 | 7.6 | 56.4 | 70.1 | 89.1 | 68.4 | 16.0 | 0 |
|  | *0.0* | *0.0* | *2.2* | *23.4* | *19.8* | *19.1* | *22.0* | *9.4* | *0.0* |
| *Spirorbis borealis* Daudin | 0 | 0 | 0 | 0 | 0 | 0 | 0 | 0 | 0 |
|  | *0.0* | *0.0* | *0.0* | *0.0* | *0.0* | *0.0* | *0.0* | *0.0* | *0.0* |
| **Arthropoda** |  |  |  |  |  |  |  |  |  |
| **Arachnida** |  |  |  |  |  |  |  |  |  |
| Halacaridae | 0 | 0 | 20.9 | 0 | 0 | 132.3 | 254.5 | 321.7 | 151.7 |
|  | *0.0* | *0.0* | *6.9* | *0.0* | *0.0* | *22.1* | *23.4* | *22.8* | *19.7* |
| **Crustacea** |  |  |  |  |  |  |  |  |  |
| *Ampithoe rubricata* Montagu | 0 | 0 | 111.3 | 9.0 | 0 | 0 | 0 | 0 | 56.8 |
|  | *0.0* | *0.0* | *22.8* | *2.7* | *0.0* | *0.0* | *0.0* | *0.0* | *18.6* |
| *Calliopius laeviusculus* Kroyer | 113.2 | 115.7 | 640.1 | 131.4 | 251.4 | 1110.8 | 283.1 | 608.7 | 335.0 |
|  | *35.8* | *13.8* | *29.9* | *16.0* | *26.7* | *71.7* | *66.2* | *68.5* | *44.2* |
| *Caprella linearis* Linnaeus | 121.2 | 120.8 | 133.7 | 95.5 | 18.7 | 14.0 | 11.9 | 120.7 | 1109.9 |
|  | *34.5* | *19.9* | *20.0* | *20.6* | *9.1* | *9.0* | *6.1* | *22.3* | *112.8* |
| *Caprella septentrionalis* Kroyer | 135.7 | 251.1 | 49.9 | 45.7 | 0 | 0 | 0 | 11.1 | 101.6 |
|  | *53.6* | *56.6* | *21.7* | *19.3* | *0.0* | *0.0* | *0.0* | *6.2* | *28.2* |
| Copepoda* | 5092.2 | 20359.1 | 27264.4 | 34738.8 | 22021.1 | 28721.0 | 36720.5 | 3507.2 | 22992.0 |
|  | *1015.5* | *4568.8* | *7411.4* | *7865.6* | *3566.3* | *15899.9* | *9448.7* | *1122.2* | *7899.5* |
| *Gammarellus angulosus* Rathke | 0 | 55.2 | 33.0 | 20.9 | 11.2 | 67.4 | 6.9 | 0 | 0 |
|  | *0.0* | *25.5* | *18.7* | *11.1* | *4.6* | *28.8* | *4.0* | *0.0* | *0.0* |
| *Gammarus oceanicus* Segerstråle | 0 | 0 | 40.7 | 5.1 | 14.9 | 21.5 | 68.0 | 12.7 | 401.8 |
|  | *0.0* | *0.0* | *17.3* | *3.9* | *10.0* | *12.5* | *22.8* | *5.5* | *64.4* |
| *Gammarus setosus* Dementieva | 0 | 0 | 9.9 | 0 | 0 | 0 | 0 | 10.7 | 0 |
|  | *0.0* | *0.0* | *2.2* | *0.0* | *0.0* | *0.0* | *0.0* | *3.6* | *0.0* |
| *Hyas sp.** | 0 | 0 | 0 | 0 | 0 | 0 | 0 | 0 | 0 |
|  | *0.0* | *0.0* | *0.0* | *0.0* | *0.0* | *0.0* | *0.0* | *0.0* | *0.0* |
| *Idotea baltica* Pallas | 0 | 10.0 | 13.7 | 1.4 | 115.0 | 129.6 | 66.9 | 67.8 | 201.4 |
|  | *0.0* | *3.4* | *4.7* | *0.8* | *22.0* | *17.8* | *19.6* | *19.5* | *22.7* |
| *Ischyrocerus anguipes* Kroyer | 1254.4 | 246.0 | 749.8 | 957.2 | 169.7 | 491.2 | 144.4 | 231.4 | 899.0 |
|  | *160.4* | *68.4* | *211.3* | *345.7* | *40.9* | *33.5* | *26.5* | *27.4* | *123.8* |
| *Leptocheirus pinguis* Stimpson | 0 | 0 | 0 | 0 | 110.2 | 251.5 | 13.4 | 85.7 | 353.3 |
|  | *0.0* | *0.0* | *0.0* | *0.0* | *20.7* | *23.6* | *9.0* | *21.4* | *35.5* |
| *Munna sp.* | 0 | 0 | 0 | 0 | 0 | 0 | 59.3 | 48.9 | 270.5 |
|  | *0.0* | *0.0* | *0.0* | *0.0* | *0.0* | *0.0* | *0.0* | *22.8* | *23.1* |
| *Pontogeneia inermis* Kroyer | 0 | 0 | 428.7 | 194.5 | 460.0 | 202.8 | 4.1 | 0 | 199.7 |
|  | *0.0* | *0.0* | *59.8* | *60.1* | *66.5* | *45.8* | *2.0* | *0.0* | *45.7* |
| *Stenothoe brevicornis* G.O. Sars | 0 | 0 | 151.0 | 151.3 | 301.1 | 51.2 | 20.7 | 9.8 | 85.4 |
|  | *0.0* | *0.0* | *34.7* | *32.6* | *29.6* | *14.0* | *10.3* | *2.7* | *25.6* |
| **Pycnogonida** |  |  |  |  |  |  |  |  |  |
| *Phoxichilidium femoratum* Rathke | 0 | 0 | 0 | 0 | 0 | 0 | 0 | 0 | 1704.6 |
|  | *0.0* | *0.0* | *0.0* | *0.0* | *0.0* | *0.0* | *0.0* | *0.0* | *523.5* |
| **Chordata** |  |  |  |  |  |  |  |  |  |
| **Actinopterygii** |  |  |  |  |  |  |  |  |  |
| *Cyclopterus lumpus* Linnaeus* | 0 | 0 | 0 | 0 | 0 | 0 | 0 | 0 | 0 |
|  | *0.0* | *0.0* | *0.0* | *0.0* | *0.0* | *0.0* | *0.0* | *0.0* | *0.0* |
| *Liparis inquilinus* Able* | 0 | 0 | 0 | 0 | 0 | 0 | 0 | 0 | 0 |
|  | *0.0* | *0.0* | *0.0* | *0.0* | *0.0* | *0.0* | *0.0* | *0.0* | *0.0* |
| *Pholis gunnellus* Linnaeus* | 0 | 0 | 0 | 0 | 0 | 0 | 1.8 | 0 | 0 |
|  | *0.0* | *0.0* | *0.0* | *0.0* | *0.0* | *0.0* | *0.2* | *0.0* | *0.0* |
| Egg masses (unknown species) | 0 | 5.1 | 11.9 | 21.7 | 51.6 | 0 | 0 | 0 | 0 |
|  | *0.0* | *2.2* | *3.4* | *17.8* | *16.5* | *0.0* | *0.0* | *0.0* | *0.0* |
| **Cnidaria** |  |  |  |  |  |  |  |  |  |
| **Hydrozoa** |  |  |  |  |  |  |  |  |  |
| *Obelia geniculata* Linnaeus** | 0 | 0 | 0 | 0 | 0 | 0 | 10.9 | 0 | 0 |
|  | *0.0* | *0.0* | *0.0* | *0.0* | *0.0* | *0.0* | *3.3* | *0.0* | *0.0* |
| **Scyphozoa** |  |  |  |  |  |  |  |  |  |
| *Haliclystus salpinx* James-Clark | 0 | 0 | 0 | 0 | 0 | 0 | 43.8 | 0 | 0 |
|  | *0.0* | *0.0* | *0.0* | *0.0* | *0.0* | *0.0* | *15.4* | *0.0* | *0.0* |
| **Echinodermata** |  |  |  |  |  |  |  |  |  |
| **Asteroidea** |  |  |  |  |  |  |  |  |  |
| *Asterias sp.* | 0 | 10.1 | 0 | 0 | 0 | 0 | 0 | 0 | 59.6 |
|  | *0.0* | *2.8* | *0.0* | *0.0* | *0.0* | *0.0* | *0.0* | *0.0* | *16.6* |
| **Ophiuroidea** |  |  |  |  |  |  |  |  |  |
| *Ophiopholis aculeata* Linnaeus | 0 | 0 | 0 | 5.0 | 32.5 | 0 | 0 | 0 | 0 |
|  | *0.0* | *0.0* | *0.0* | *2.4* | *12.7* | *0.0* | *0.0* | *0.0* | *0.0* |
| **Ectoprocta** |  |  |  |  |  |  |  |  |  |
| **Gymnolaemata** |  |  |  |  |  |  |  |  |  |
| *Electra pilosa* Linnaeus** | 22.5 | 46.5 | 33.5 | 46.2 | 54.1 | 66.8 | 70.1 | 46.3 | 77.8 |
|  | *7.6* | *10.0* | *6.7* | *10.1* | *13.8* | *12.7* | *13.9* | *19.0* | *21.1* |
| **Stenolaemata** |  |  |  |  |  |  |  |  |  |
| *Lichenopora sp*.** | 0 | 0 | 0 | 0 | 0 | 0 | 0 | 0 | 0 |
|  | *0.0* | *0.0* | *0.0* | *0.0* | *0.0* | *0.0* | *0.0* | *0.0* | *0.0* |
| **Mollusca** |  |  |  |  |  |  |  |  |  |
| **Bivalvia** |  |  |  |  |  |  |  |  |  |
| *Hiatella arctica* Linnaeus | 0 | 0 | 0 | 0 | 0 | 0 | 0 | 0 | 61.3 |
|  | *0.0* | *0.0* | *0.0* | *0.0* | *0.0* | *0.0* | *0.0* | *0.0* | *7.2* |
| *Modiolus modiolus* Linnaeus | 0 | 0 | 50.8 | 26.3 | 32.5 | 12.6 | 58.0 | 0 | 0 |
|  | *0.0* | *0.0* | *13.8* | *15.2* | *8.7* | *6.3* | *7.9* | *0.0* | *0.0* |
| *Mytilus sp.* | 178.9 | 257.2 | 346.0 | 165.7 | 205.7 | 122.1 | 74.3 | 204694.4 | 238465.9 |
|  | *28.8* | *49.7* | *101.2* | *133.8* | *97.5* | *77.3* | *25.4* | *1685.5* | *7985.6* |
| **Gastropoda** |  |  |  |  |  |  |  |  |  |
| *Dendronotus frondosus* Ascanius | 0 | 134.9 | 0 | 0 | 0 | 0 | 0 | 0 | 0 |
|  | *0.0* | *55.5* | *0.0* | *0.0* | *0.0* | *0.0* | *0.0* | *0.0* | *0.0* |
| *Lacuna vincta* Montagu | 2267.5 | 1287.3 | 1454.2 | 1072.5 | 436.9 | 127.5 | 1128.2 | 11401.4 | 24002.9 |
|  | *986.6* | *774.2* | *452.1* | *200.8* | *98.7* | *45.2* | *106.4* | *3011.8* | *4561.2* |
| *Margarites helicinus* Phipps | 0 | 77.8 | 25.9 | 58.3 | 97.0 | 69.1 | 78.4 | 2091.8 | 259.7 |
|  | *0.0* | *21.1* | *13.2* | *11.5* | *95.5* | *46.4* | *66.2* | *106.9* | *49.9* |
| Egg masses (*L. vincta*) | 0 | 0 | 13.9 | 18.9 | 9.7 | 73.7 | 51.9 | 0 | 0 |
|  | *0.0* | *0.0* | *2.2* | *1.6* | *2.6* | *46.6* | *16.8* | *0.0* | *0.0* |
| **Polyplacophora** |  |  |  |  |  |  |  |  |  |
| *Tonicella marmorea* Fabricius | 0 | 31.8 | 0 | 0 | 0 | 0 | 0 | 0 | 0 |
|  | *0.0* | *22.8* | *0.0* | *0.0* | *0.0* | *0.0* | *0.0* | *0.0* | *0.0* |
| **Nemertea** |  |  |  |  |  |  |  |  |  |
| **Enopla** |  |  |  |  |  |  |  |  |  |
| *Tetrastemma sp.** | 0 | 8.0 | 19.8 | 72.3 | 48.0 | 11.6 | 2.7 | 0 | 0 |
|  | *0.0* | *0.1* | *2.7* | *9.3* | *10.7* | *0.5* | *0.1* | *0.0* | *0.0* |
|  |  |  |  |  |  |  |  |  |  |

***Agarum clathratum***

|  |  |  |  |  |  |  |  |  |  |
| --- | --- | --- | --- | --- | --- | --- | --- | --- | --- |
| **Taxon** | **Feb** | **Mar** | **Apr** | **May** | **Jun** | **Jul** | **Aug** | **Sep** | **Oct** |
| **Annelida** |  |  |  |  |  |  |  |  |  |
| **Polychaeta** |  |  |  |  |  |  |  |  |  |
| *Alitta virens* M Sars | 39.6 | 4.2 | 0 | 0 | 0 | 0 | 0 | 0 | 0 |
|  | *8.2* | *0.9* | *0.0* | *0.0* | *0.0* | *0.0* | *0.0* | *0.0* | *0.0* |
| *Autolytinae sp.* | 0 | 0 | 0 | 0 | 0 | 0 | 6.4 | 0 | 0 |
|  | *0.0* | *0.0* | *0.0* | *0.0* | *0.0* | *0.0* | *2.2* | *0.0* | *0.0* |
| *Bylgides sarsi* Kinberg in Malmgren | 0 | 0 | 0 | 0 | 0 | 0 | 6.4 | 0 | 0 |
|  | *0.0* | *0.0* | *0.0* | *0.0* | *0.0* | *0.0* | *5.1* | *0.0* | *0.0* |
| *Lepidonotus squamatus* Linnaeus | 44.7 | 22.3 | 2.0 | 0 | 0 | 0 | 0 | 0 | 0 |
|  | *16.1* | *6.0* | *0.7* | *0.0* | *0.0* | *0.0* | *0.0* | *0.0* | *0.0* |
| *Nereis pelagica* Linnaeus | 50.8 | 91.1 | 0 | 0 | 0 | 0 | 0 | 0 | 0 |
|  | *9.7* | *10.3* | *0.0* | *0.0* | *0.0* | *0.0* | *0.0* | *0.0* | *0.0* |
| *Phyllodoce mucosa* Örsted | 0 | 0 | 0 | 0 | 0 | 0 | 0 | 0 | 0 |
|  | *0.0* | *0.0* | *0.0* | *0.0* | *0.0* | *0.0* | *0.0* | *0.0* | *0.0* |
| *Spirorbis borealis* Daudin | 40.5 | 95.8 | 69.9 | 165.3 | 251.1 | 233.7 | 194.5 | 3073.6 | 2751.1 |
|  | *8.8* | *16.4* | *20.1* | *21.3* | *44.7* | *39.8* | *66.2* | *468.4* | *455.0* |
| **Arthropoda** |  |  |  |  |  |  |  |  |  |
| **Arachnida** |  |  |  |  |  |  |  |  |  |
| Halacaridae | 0 | 16.7 | 0 | 0 | 0 | 0 | 0 | 0 | 0 |
|  | *0.0* | *2.3* | *0.0* | *0.0* | *0.0* | *0.0* | *0.0* | *0.0* | *0.0* |
| **Crustacea** |  |  |  |  |  |  |  |  |  |
| *Ampithoe rubricata* Montagu | 0 | 0 | 0 | 0 | 0 | 0 | 0 | 20.2 | 22.8 |
|  | *0.0* | *0.0* | *0.0* | *0.0* | *0.0* | *0.0* | *0.0* | *9.1* | *9.0* |
| *Calliopius laeviusculus* Kroyer | 3.2 | 3.2 | 29.7 | 11.7 | 19.1 | 69.4 | 0 | 0 | 0 |
|  | *0.5* | *2.4* | *11.8* | *7.3* | *8.0* | *22.7* | *0.0* | *0.0* | *0.0* |
| *Caprella linearis* Linnaeus | 0 | 0 | 0 | 4.1 | 0 | 0 | 0 | 6.8 | 0 |
|  | *0.0* | *0.0* | *0.0* | *2.0* | *0.0* | *0.0* | *0.0* | *3.1* | *0.0* |
| *Caprella septentrionalis* Kroyer | 0 | 0 | 0 | 0 | 0 | 0 | 0 | 0 | 0 |
|  | *0.0* | *0.0* | *0.0* | *0.0* | *0.0* | *0.0* | *0.0* | *0.0* | *0.0* |
| Copepoda* | 1098.4 | 975.2 | 1210.8 | 11859.4 | 19319.9 | 4157.7 | 17118.2 | 3923.0 | 4044.2 |
|  | *163.2* | *222.7* | *298.4* | *1855.2* | *5781.1* | *465.9* | *5987.5* | *1654.1* | *990.0* |
| *Gammarellus angulosus* Rathke | 336.4 | 24.3 | 9.8 | 0 | 0 | 0 | 0 | 0 | 0 |
|  | *55.7* | *20.1* | *7.4* | *0.0* | *0.0* | *0.0* | *0.0* | *0.0* | *0.0* |
| *Gammarus oceanicus* Segerstråle | 0 | 0 | 0 | 0 | 0 | 0 | 0 | 0 | 0 |
|  | *0.0* | *0.0* | *0.0* | *0.0* | *0.0* | *0.0* | *0.0* | *0.0* | *0.0* |
| *Gammarus setosus* Dementieva | 2.8 | 4.9 | 1.2 | 0 | 0 | 0 | 0 | 0 | 0 |
|  | *0.9* | *0.7* | *0.6* | *0.0* | *0.0* | *0.0* | *0.0* | *0.0* | *0.0* |
| *Hyas sp.** | 1.0 | 3.9 | 0 | 0 | 0 | 0 | 0 | 0 | 0 |
|  | *2.2* | *0.9* | *0.0* | *0.0* | *0.0* | *0.0* | *0.0* | *0.0* | *0.0* |
| *Idotea baltica* Pallas | 0 | 0 | 0 | 0 | 0 | 0 | 0 | 0 | 0 |
|  | *0.0* | *0.0* | *0.0* | *0.0* | *0.0* | *0.0* | *0.0* | *0.0* | *0.0* |
| *Ischyrocerus anguipes* Kroyer | 0 | 241.0 | 200.2 | 161.7 | 159.6 | 625.1 | 128.7 | 201.0 | 135.1 |
|  | *0.0* | *48.5* | *36.9* | *45.8* | *63.8* | *71.1* | *52.0* | *52.4* | *43.0* |
| *Leptocheirus pinguis* Stimpson | 0 | 0 | 0 | 0 | 0 | 0 | 0 | 51.3 | 0 |
|  | *0.0* | *0.0* | *0.0* | *0.0* | *0.0* | *0.0* | *0.0* | *13.5* | *0.0* |
| *Munna sp.* | 0 | 0 | 0 | 0 | 0 | 0 | 0 | 0 | 0 |
|  | *0.0* | *0.0* | *0.0* | *0.0* | *0.0* | *0.0* | *0.0* | *0.0* | *0.0* |
| *Pontogeneia inermis* Kroyer | 0 | 54.2 | 92.9 | 31.0 | 100.7 | 143.4 | 0 | 0 | 0 |
|  | *0.0* | *14.1* | *23.6* | *19.2* | *12.8* | *16.7* | *0.0* | *0.0* | *0.0* |
| *Stenothoe brevicornis* G.O. Sars | 0.0 | 0 | 9.9 | 0.0 | 131.7 | 14.3 | 55.9 | 27.7 | 124.9 |
|  | *0.0* | *0.0* | *2.8* | *0.0* | *33.0* | *5.4* | *19.8* | *12.4* | *12.6* |
| **Pycnogonida** |  |  |  |  |  |  |  |  |  |
| *Phoxichilidium femoratum* Rathke | 0 | 0 | 0 | 0 | 0 | 0 | 0 | 0 | 0 |
|  | *0.0* | *0.0* | *0.0* | *0.0* | *0.0* | *0.0* | *0.0* | *0.0* | *0.0* |
| **Chordata** |  |  |  |  |  |  |  |  |  |
| **Actinopterygii** |  |  |  |  |  |  |  |  |  |
| *Cyclopterus lumpus* Linnaeus* | 0 | 2.4 | 5.6 | 9.8 | 0 | 0 | 0 | 0 | 0 |
|  | *0.0* | *0.8* | *2.1* | *2.3* | *0.0* | *0.0* | *0.0* | *0.0* | *0.0* |
| *Liparis inquilinus* Able* | 6.3 | 2.1 | 3.4 | 0.0 | 0 | 0 | 0 | 0 | 0 |
|  | *2.2* | *0.7* | *0.8* | *0.0* | *0.0* | *0.0* | *0.0* | *0.0* | *0.0* |
| *Pholis gunnellus* Linnaeus* | 0 | 2.5 | 0 | 0 | 0 | 2.1 | 2.8 | 0 | 0 |
|  | *0.0* | *1.2* | *0.0* | *0.0* | *0.0* | *0.9* | *2.2* | *0.0* | *0.0* |
| Egg masses (unknown species) | 0 | 0 | 0 | 0 | 0 | 0 | 0 | 0 | 0 |
|  | *0.0* | *0.0* | *0.0* | *0.0* | *0.0* | *0.0* | *0.0* | *0.0* | *0.0* |
| **Cnidaria** |  |  |  |  |  |  |  |  |  |
| **Hydrozoa** |  |  |  |  |  |  |  |  |  |
| *Obelia geniculata* Linnaeus** | 0 | 0 | 0 | 0 | 0 | 2.6 | 2.8 | 15.8 | 40.9 |
|  | *0.0* | *0.0* | *0.0* | *0.0* | *0.0* | *1.1* | *0.9* | *2.8* | *13.5* |
| **Scyphozoa** |  |  |  |  |  |  |  |  |  |
| *Haliclystus salpinx* James-Clark | 0 | 0 | 4.6 | 0 | 16.0 | 5.9 | 37.7 | 29.8 | 22.1 |
|  | *0.0* | *0.0* | *0.3* | *0.0* | *2.0* | *0.9* | *4.6* | *7.1* | *6.9* |
| **Echinodermata** |  |  |  |  |  |  |  |  |  |
| **Asteroidea** |  |  |  |  |  |  |  |  |  |
| *Asterias sp.* | 0 | 0 | 0 | 0 | 0 | 0 | 0 | 0 | 0 |
|  | *0.0* | *0.0* | *0.0* | *0.0* | *0.0* | *0.0* | *0.0* | *0.0* | *0.0* |
| **Ophiuroidea** |  |  |  |  |  |  |  |  |  |
| *Ophiopholis aculeata* Linnaeus | 0 | 0 | 0 | 0 | 0 | 0 | 21.5 | 0 | 9.2 |
|  | *0.0* | *0.0* | *0.0* | *0.0* | *0.0* | *0.0* | *0.9* | *0.0* | *2.3* |
| **Ectoprocta** |  |  |  |  |  |  |  |  |  |
| **Gymnolaemata** |  |  |  |  |  |  |  |  |  |
| *Electra pilosa* Linnaeus** | 0 | 9.8 | 0 | 0 | 0 | 53.8 | 101.9 | 298.3 | 238.1 |
|  | *0.0* | *2.2* | *0.0* | *0.0* | *0.0* | *11.5* | *20.7* | *46.5* | *40.7* |
| **Stenolaemata** |  |  |  |  |  |  |  |  |  |
| *Lichenopora sp*.** | 63.5 | 86.8 | 76.3 | 50.9 | 12.4 | 113.5 | 277.8 | 191.9 | 4088.9 |
|  | *13.8* | *22.2* | *13.9* | *10.1* | *0.9* | *41.8* | *33.7* | *34.6* | *108.8* |
| **Mollusca** |  |  |  |  |  |  |  |  |  |
| **Bivalvia** |  |  |  |  |  |  |  |  |  |
| *Hiatella arctica* Linnaeus | 0 | 0 | 0 | 0 | 0 | 0 | 0 | 0 | 0 |
|  | *0.0* | *0.0* | *0.0* | *0.0* | *0.0* | *0.0* | *0.0* | *0.0* | *0.0* |
| *Modiolus modiolus* Linnaeus | 29.9 | 43.2 | 17.6 | 67.3 | 27.4 | 28.8 | 137.5 | 8.1 | 0 |
|  | *2.8* | *2.8* | *3.7* | *16.9* | *13.4* | *16.8* | *46.5* | *0.2* | *0.0* |
| *Mytilus sp.* | 102.0 | 143.4 | 7.8 | 45.8 | 50.4 | 68.7 | 16.7 | 381.2 | 454.2 |
|  | *12.9* | *14.1* | *0.4* | *10.8* | *8.7* | *8.3* | *2.4* | *51.6* | *49.8* |
| **Gastropoda** |  |  |  |  |  |  |  |  |  |
| *Dendronotus frondosus* Ascanius | 0 | 0 | 0 | 0 | 0 | 0 | 0 | 0 | 75.0 |
|  | *0.0* | *0.0* | *0.0* | *0.0* | *0.0* | *0.0* | *0.0* | *0.0* | *13.2* |
| *Lacuna vincta* Montagu | 54.8 | 1104.0 | 260.2 | 178.0 | 444.2 | 109.2 | 435.4 | 7246.1 | 10496.6 |
|  | *12.8* | *765.1* | *46.8* | *66.0* | *169.2* | *59.6* | *44.7* | *1010.5* | *2337.4* |
| *Margarites helicinus* Phipps | 33.4 | 45.5 | 35.4 | 28.1 | 39.7 | 19.6 | 9.4 | 281.6 | 30.7 |
|  | *8.1* | *12.3* | *13.4* | *9.5* | *10.5* | *4.2* | *1.6* | *44.8* | *13.2* |
| Egg masses (*L. vincta*) | 5.0 | 0 | 0 | 70.6 | 76.9 | 43.8 | 28.3 | 10.8 | 247.2 |
|  | *2.2* | *0.0* | *0.0* | *21.0* | *21.3* | *19.1* | *5.8* | *2.5* | *46.7* |
| **Polyplacophora** |  |  |  |  |  |  |  |  |  |
| *Tonicella marmorea* Fabricius | 0 | 13.1 | 0 | 0 | 0 | 0 | 0 | 0 | 0 |
|  | *0.0* | *2.2* | *0.0* | *0.0* | *0.0* | *0.0* | *0.0* | *0.0* | *0.0* |
| **Nemertea** |  |  |  |  |  |  |  |  |  |
| **Enopla** |  |  |  |  |  |  |  |  |  |
| *Tetrastemma sp.** | 0 | 0 | 0 | 0 | 0 | 0 | 0 | 0 | 0 |
|  | *0.0* | *0.0* | *0.0* | *0.0* | *0.0* | *0.0* | *0.0* | *0.0* | *0.0* |

* Denotes taxon excluded from MDS, ANOSIM, and SIMPER analyses.

** Denotes colonial taxon in which each colony was counted as one individual.
